# Including Stable Carbon Isotopes to Evaluate the Dynamics of Soil Carbon in the Land‐Surface Model ORCHIDEE

**DOI:** 10.1029/2018MS001392

**Published:** 2019-11-17

**Authors:** Marta Camino‐Serrano, Marwa Tifafi, Jérôme Balesdent, Christine Hatté, Josep Peñuelas, Sophie Cornu, Bertrand Guenet

**Affiliations:** ^1^ CREAF, Universitat Autònoma de Barcelona Catalonia Spain; ^2^ CSIC, Global Ecology Unit CREAF‐CSIC‐UAB Catalonia Spain; ^3^ Laboratoire des Sciences du Climat et de l'Environnement, LSCE/IPSL, CEA‐CNRS‐UVSQ Université Paris‐Saclay Paris France; ^4^ CNRS, IRD, INRA, Coll France, CEREGE, Aix Marseille Univ Aix en Provence France

**Keywords:** soil organic carbon, stable isotopes, land surface model

## Abstract

Soil organic carbon (SOC) is a crucial component of the terrestrial carbon cycle and its turnover time in models is a key source of uncertainty. Studies have highlighted the utility of δ^13^C measurements for benchmarking SOC turnover in global models. We used ^13^C as a tracer within a vertically discretized soil module of a land‐surface model, Organising Carbon and Hydrology In Dynamic Ecosystems‐ Soil Organic Matter (ORCHIDEE‐SOM). Our new module represents some of the processes that have been hypothesized to lead to a ^13^C enrichment with soil depth as follows: 1) the Suess effect and CO_2_ fertilization, 2) the relative ^13^C enrichment of roots compared to leaves, and 3) ^13^C discrimination associated with microbial activity. We tested if the upgraded soil module was able to reproduce the vertical profile of δ^13^C within the soil column at two temperate sites and the short‐term change in the isotopic signal of soil after a shift in C3/C4 vegetation. We ran the model over Europe to test its performance at larger scale. The model was able to simulate a shift in the isotopic signal due to short‐term changes in vegetation cover from C3 to C4; however, it was not able to reproduce the overall vertical profile in soil δ^13^C, which arises as a combination of short and long‐term processes. At the European scale, the model ably reproduced soil CO_2_ fluxes and total SOC stock. These findings stress the importance of the long‐term history of land cover for simulating vertical profiles of δ^13^C. This new soil module is an emerging tool for the diagnosis and improvement of global SOC models.

## Key Points

- Soil ^13^C was incorporated as a tracer in the land surface model ORCHIDEE‐SOM
- The model was able to reproduce short‐term changes such as those observed after a shift in C3/C4 vegetation
- Long‐term changes in δ^13^C were more difficult to represent, highlighting the importance of some missing mechanisms

## 1 Introduction

Global soil organic carbon (SOC) simulated by Earth system models (ESMs) may be consistent with global observations, but the uncertainty surrounding models output remains collectively high (Huntzinger et al., 2017; Todd‐Brown et al., 2013). Good overall model‐data agreement may result from compensating model biases, and unless the sources of these biases are identified and corrected, ESMs stand little chance of providing useful projections of SOC under a changing climate (Todd‐Brown et al., 2013). Reducing the high uncertainty associated with predictions of SOC in ESMs involves a range of approaches, including the incorporation of missing key carbon cycling mechanisms and improving model parameterization.

The turnover times of modeled soil‐carbon pools are key sources of uncertainty in the simulations of SOC, primarily because experimentally isolating the SOC pools as defined by ESMs is technically and conceptually difficult (Barré et al., 2010; Elliott et al., 1996). In the long‐term, we may use innovative approaches that radically change the soil carbon modules of ESMs (Abramoff et al., 2017; Huang et al., 2018; Luo et al., 2016) but such approaches would first require a deep rethinking of the ESMs themselves. In the short‐term, we may use C‐tracers to better constrain the model parameters, which is something already being done in site‐scale models (Braakhekke et al., 2014). Using observational isotopic data to constrain soil turnover times is not yet possible for ESMs, because global models usually do not represent either stable carbon or radiocarbon isotopes even though some attempts have been carried out with ORCHIDEE for radiocarbon (Tifafi et al., 2018). The Community Land Model (CLM) has fully implemented photosynthetic discrimination, and tracks δ^13^C within the biomass and soil carbon but without post‐photosynthetic discrimination (Koven et al., 2013). Nevertheless, radiocarbon has been used for decades to constrain SOC turnover times in a 1‐D model (Coleman et al., 1997) and has been used to indirectly evaluate ESMs (He et al., 2016). Chen et al. (2019) recently used radiocarbon to evaluate the E3SM ESMs, and they underline the importance of fresh carbon supply on soil carbon turnover including in deep soils. Stable isotope tracers (^13^C) have also been considered to be a potential tool for constraining SOC turnover times in the medium‐long term (decades to century) in global models (Balesdent et al., 2018a). To the best of our knowledge, stable isotope tracers have not yet been applied.

Observed variations of the composition of stable carbon isotopes (δ^13^C) are useful for tracing carbon sources and fluxes between plants, microorganisms, and soils, thus serving to constrain various processes and identify carbon sources (Balesdent & Mariotti, 1987; Ehleringer et al., 2000). Including δ^13^C in global carbon models can therefore help us to better understand the cycling of soil carbon, diagnose model imperfections, and improve model parameterization. However, variations in the isotopic composition of soils remain difficult to predict (Högberg et al., 2005) because environmental factors impact discrimination during photosynthesis and C allocation in the plants (Brüggemann et al., 2011). In addition, the mechanisms underlying discrimination of stable isotopes aboveground (e.g., photosynthetic discrimination; Bowling et al., 2008) have not been fully disentangled.

**Figure 1:**
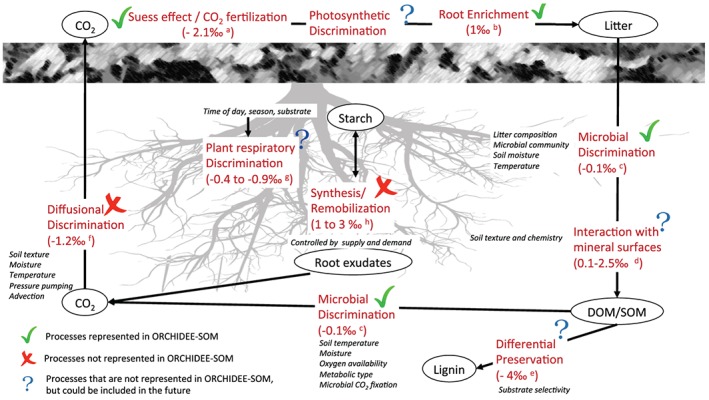
Overview of the processes and factors determining the isotopic signature of soil C pools and fluxes (modified from Brüggemann et al., 2011). Oval boxes represent C pools, processes are in red and the controlling factors of each process are in italics. Discrimination values are taken from (^a^) Keeling et al. (2017), (^b^) Hobbie and Werner (2004), (^c^) Jagercikova et al. (2017), (^d^) Bird et al. (2003), (^e^) Gleixner et al. (2005), (^f^) Yakir and Wang (1996), (^g^) Klumpp et al. (2005), and (^h^) Brugnoli et al. (1988).

The long‐term reconstruction of ecosystem dynamics, more specifically the historical changes of relative C3/C4 vegetation, is one of the most common applications of stable isotopes. Based on observations showing that photosynthetic ^13^C discrimination is large compared to post‐photosynthetic discrimination (Bowling et al., 2008), models using δ^13^C for the reconstruction of ecosystem dynamics consider post‐photosynthetic discrimination as negligible (e.g. Diels et al., 2004; Molina et al., 2001; Raczka et al., 2016). Nevertheless, other mechanisms driving the δ^13^C in soil may also significantly affect the observed values. Indeed, δ^13^C tends to increase with soil depth across many ecosystems, with widespread observations of 1–3‰ enrichment in δ^13^C in the soil profiles of forests (Balesdent et al., 1993; Bird et al., 2003; Brüggemann et al., 2011; Ehleringer et al., 2000). The processes involved in the variations of SOC isotopic values are diverse and cover large spatial and temporal scales (Figure 1).

The potential mechanisms for the natural variation (i.e., all except Suess effect—the depletion of atmospheric and plant‐derived ^13^C due to the increase in fossil‐fuel emissions in the 20th century—and CO_2_ fertilization in Figure 1) of stable isotopes in soil can be summarized into three groups as follows: physical mixing, preferential preservation, and microbial discrimination (Ehleringer et al., 2000). First, the physical mixing of bulk SOC derived from biomass with different isotopic signatures may partly account for the differences in isotopic signatures in the SOC pool. For example, mixing between C3 and C4 plants, or between leaf litter and roots, have different isotopic composition. Second, the preferential preservation or movement of some compounds may cause a shift in the isotopic signal of SOC. For example, lignin is depleted in ^13^C more than bulk SOC, so SOC in ecosystems richer in lignin will have a lower δ^13^C (Ehleringer et al., 2000; Gleixner et al., 2005; Lichtfouse et al., 1995, 1998). Third, microbial discrimination of carbon isotopes during the decomposition of SOC may enrich the substrate by a differential use of ^13^C‐depleted carbon sources in the metabolic reactions of litter and SOC decomposition. The evidence for microbial kinetic discrimination, however, is inconsistent across studies (Ehleringer et al., 2000; Wynn et al., 2006), and ^13^C enrichment of respired CO_2_ compared to the bulk substrate for C3 soils suggests the opposite to what we expect by assuming a kinetic discrimination during SOC decomposition (Werth & Kuzyakov, 2010). The ^13^C enrichment in soil microorganisms may be due to the preferential use of ^13^C‐enriched compounds, such as sugars or cellulose, by microbial decomposers (Menichetti et al., 2015; Werth & Kuzyakov, 2010). In summary, no consensus has been reached on the relative importance of each process to the observed variation in soil ^13^C. Moreover, δ^13^C of SOC is also indirectly impacted by the depletion of ^13^C in relatively recently generated atmospheric CO_2_ due to the burning of fossil fuels (depleted in ^13^C‐the Suess effect; Högberg et al., 2005; Keeling et al., 2017; Tans et al., 1979; Wynn et al., 2006).

ORCHIDEE‐SOM is a recently‐developed soil module within the land‐surface model ORCHIDEE that represents a substantial advance toward accurate future predictions of global soil carbon fluxes by modeling deep, soil layer‐discretized SOC to 2 m (Camino‐Serrano et al., 2018). However, the model in still in early stages of its development, with attendant uncertainty in both its output and their interpretation, for which data‐based stable isotope parameter calibration will help to constrain the parameters involved in carbon transfers and SOC turnover times (Luo et al., 2016). Our aim was therefore to incorporate ^13^C into ORCHIDEE‐SOM and to test if the upgraded model could reproduce the stock of ^13^C in the following two cases: 1) observed stock of ^13^C at two temperate sites, a forest and a grassland and 2) the short‐term change in the isotopic signal of soil after a C3/C4 shift in vegetation at two temperate experimental sites where C4 crops were planted in C3 soils a few decades ago. The benefits of introducing soil ^13^C into ORCHIDEE‐SOM are twofold as follows: 1) it will give us insight into the factors controlling the dynamics of ^13^C in soils and 2) it will allow us to use another source of data to evaluate the model.

## 2 Materials and Methods

### 2.1 The ORCHIDEE‐SOM Model: The Starting Point

ORCHIDEE‐SOM is an extension of the soil module in ORCHIDEE, the global land‐surface component of the Institut Pierre‐Simon Laplace ESM. ORCHIDEE‐SOM starts from the default structure of the ORCHIDEE soil model, based on the CENTURY model (Parton et al., 1987) that represents four litter (metabolic and structural below‐ and above‐ ground litter) and three SOC (passive, slow, and active) pools distinguished by their turnover times. Two pools were added to represent dissolved organic carbon (DOC) and labeled stable and labile DOC with low and high turnover times, respectively. The values of the decomposition parameters are based on Table 1 from Camino‐Serrano et al. (2018). In this study, we did not perform any formal data integration due to the computational resources required to run the model. The soil profile is discretized into 11 layers of geometrically increasing thicknesses to a depth of 2 m (Campoy et al., 2013). The biological processing of litter, SOC and DOC, and the sorption of DOC to minerals are represented within each layer. Soil carbon is transported between layers due to the vertical advection of DOC and the diffusion of SOC and DOC. In the following subsections, we describe the processes included in ORCHIDEE‐SOM in more detail. Camino‐Serrano et al. (2018) have provided an exhaustive description of the model and some clarifying details are given in the supporting information.

**Table 1:** Factors Affecting SOM δ^13^C and Their Use in the Model ORCHIDEE‐SOM

Factor	Magnitude (approx.)	In ORCHIDEE‐SOM
1. Suess effect (including changes in δ^13^C─CO_2_ in the atmosphere and the effect of increasing atmospheric CO_2_ concentration on δ^13^C‐litter)	Transient (see Fig. 2)	Prepare a temporal record of litter δ^13^C inputs with ^13^C depletion for 1765–2011
2. Difference in the isotopic composition between roots and plant leaves/litter	+1‰	Introduce a parameter to enrich input δ^13^C in “belowground” litter (assumed as roots) compared to “aboveground” litter (assumed as leaves)
3. Isotopic fractionation associated with heterotrophic respiration	CO_2_ depleted by 0.1‰ compared to substrate.	Introduce a discrimination factor in the fluxes associated with heterotrophic respiration

#### 2.1.1 Biological Activity

The decomposition of litter, SOC, and DOC are represented in ORCHIDEE‐SOM following first‐order kinetic equations, with different decomposition rates for each pool parameterized by Camino‐Serrano et al. (2018). All decomposed litter and SOC enter the DOC pool, which is then partly respired and partly returned to the original SOC pools. Modeling microbial biomass as a separate soil pool is key for improving the representation of soil carbon in models (Schmidt et al., 2011).

#### 2.1.2 Sorption to Minerals

Sorption and organo‐mineral interactions are usually neglected in global models, such as ORCHIDEE, despite their large influence on SOC stabilization (Kalbitz et al., 2005; Schrumpf et al., 2013). In contrast, ORCHIDEE‐SOM represents the adsorption of DOC to soil minerals using an equilibrium‐partition coefficient (Nodvin et al., 1986), which implies that the DOC is split into two pools as follows: free DOC in soil solution and DOC adsorbed on soil minerals. This approach assumes that the equilibrium between adsorption and desorption is very rapid in comparison to other processes, which is supported by previous work (Kothawala et al., 2008). The remaining DOC free in solution can be transported between soil layers with the flux of soil water, whereas the adsorbed DOC is immobilized and therefore immune to decomposition.

#### 2.1.3 Carbon Transport Through the Soil

ORCHIDEE‐SOM accounts for the diffusive and advective fluxes that lead to the reallocation of soil carbon along the vertical profile. The pools of belowground litter, SOC, and DOC are subject to diffusion. Diffusion is represented by Fick's second law, which is a proxy for bioturbation in the case of SOC whereas it is stricto sensu diffusive for DOC transport (Camino‐Serrano et al., 2018). We used a diffusion coefficient that varies as a function of soil depth (Jagercikova et al., 2014), which diverges from the original ORCHIDEE‐SOM version that uses a constant diffusion coefficient across soil depths. We based this decision on results from another study that found an improvement of the simulated SOC stocks and SOC age profiles in ORCHIDEE‐SOM using the depth‐dependent diffusion coefficient (Tifafi et al., 2018). Finally, DOC in only the soluble pools are subject to transport with the soil‐water flux, that is, by advection. Advective fluxes of DOC are calculated by simply multiplying the DOC concentration in each layer by the water flux out of that layer (Futter et al., 2007).

### 2.2 Stable Carbon Isotope (^13^C) as a Tracer in Soil

ORCHIDEE‐SOM simulates all the processes mentioned above for total carbon. Here, we sought to simulate the observed ^13^C stock as a soil tracer for all soil carbon pools, including the vertical distribution of ^13^C. The inputs of ^13^C in our model come directly from a predefined δ^13^C value given to litter, meaning that ORCHIDEE‐SOM does not calculate the ^13^C fraction in living vegetation. We choose such an approach to reduce uncertainty of δ^13^C due to biases in modeled vegetation processes (allocation, phenology, etc.). However, doing so neglects potentially large changes in photosynthetic discrimination due to changes in climate impacting leaf stomatal conductance.

The various terrestrial carbon compartments (soil carbon input, litter, SOC, DOC, and heterotrophic respiration) are represented in the original version of ORCHIDEE‐SOM as a system of interacting matrices, with a single dimension referring to total carbon. We therefore added a new dimension containing the ^13^C stock for each carbon compartment and pool, calculated as a function of the bulk soil carbon. The ^13^C/^12^C isotopic ratio of litter (*R*[sample]) was calculated based on the classical equation by O'Leary (1981):

(1) $$\delta^{13}C\;\left(‰\right)=\left[\frac{R\left(\text{sample}\right)}{R\left(\text{standard}\right)}-1\right],$$

where δ^13^C is the input δ^13^C of the litter, *R* (standard) is the ^13^C/^12^C isotopic ratio of the standard (0.0111802 for the standard in general use, Vienna‐Pee Dee Belemnite (V‐PDB)), and *R* (sample) is the ^13^C/^12^C isotopic ratio of the litter. *R* (standard) and the input litter δ^13^C are known values, so we applied equation (1) to calculate the ^13^C/^12^C ratio of litter (*R*[sample]). The ^13^C/^12^C ratio was then used to calculate ^13^C stock as a function of the total soil carbon stock (^12^C + ^13^C stocks, assuming ^14^C stock was negligible):

(2) LitterC13=Rsample1+Rsample×LitterCTotal,

where Litter(^13^C) is the litter ^13^C stock (g C m^−2^) and Litter (C_Total_) is the total soil carbon stock (g C m^−2^). All processes that affect total soil carbon in litter, SOC and DOC (including decomposition, heterotrophic respiration, diffusion, and advection) in the model also apply to ^13^C. The current version of the model consequently calculates soil ^13^C stocks and fluxes alongside the total carbon stocks and fluxes.

The simulated δ^13^C was calculated back within the model from the simulated ^13^C stocks and the total carbon stocks using equations (1) and (2) to facilitate the comparison of model measurements. The output δ^13^C was calculated for total soil carbon, that is, the sum of the total litter and soil carbon pools and for each carbon pool separately: total soil carbon, passive carbon, slow carbon, active carbon, metabolic belowground litter, structural belowground litter, and DOC.

### 2.3 Sources of variation of soil ^13^C

The measured δ^13^C in soil is not constant but tends to increase with depth (Ehleringer et al., 2000; Garten et al., 2008). We designed a modeling strategy for the four main potential mechanisms underlying this increase, when possible, as explained below.

#### 2.3.1 The Suess and pCO_2_ Effects

**Figure 2:**
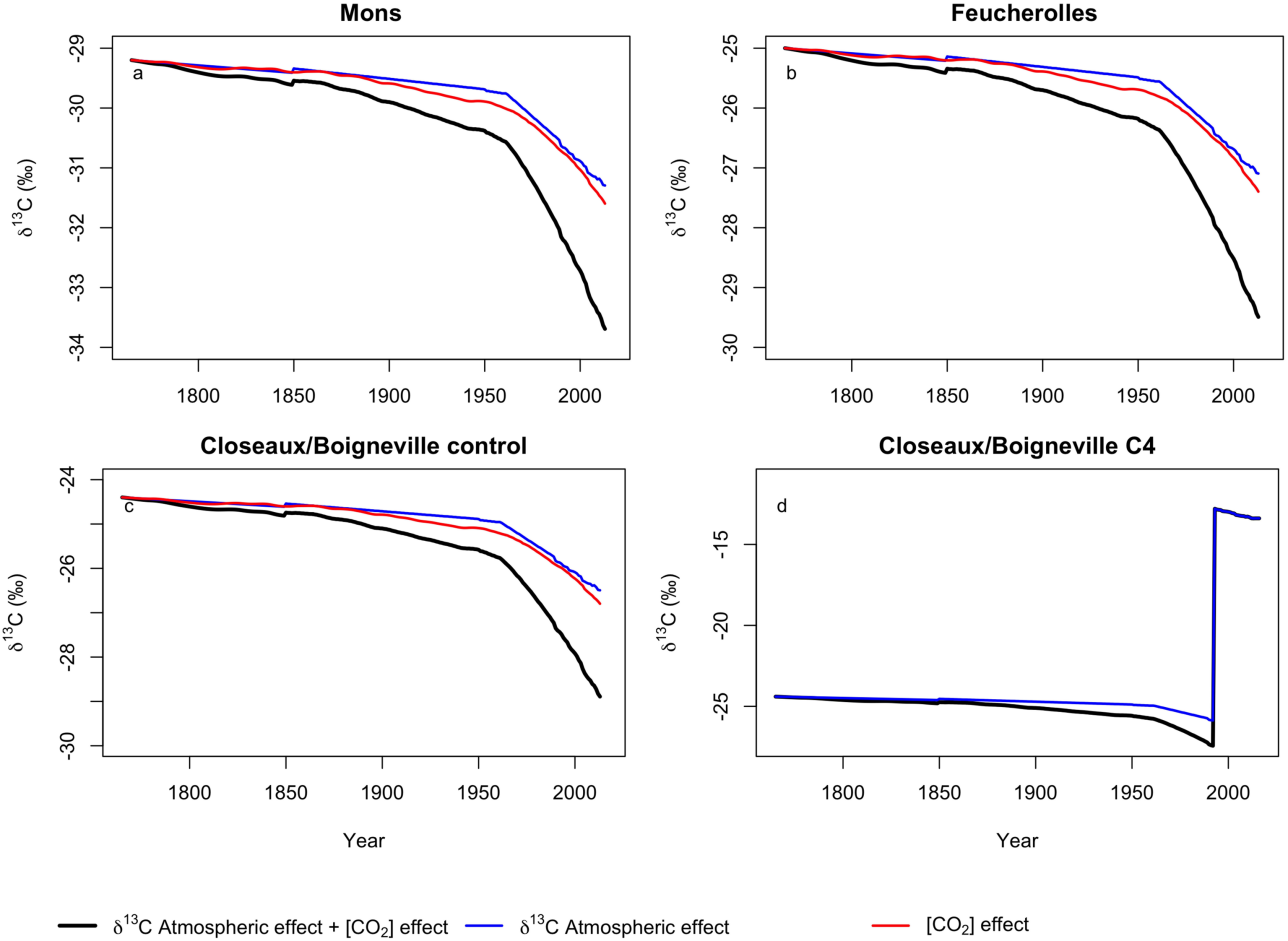
Synthetic time series of litter δ^13^C for (a) Feucherolles, (b) Mons, (c) Closeaux and Boigneville control experiment, and (d) Closeaux and Boigneville C4 experiment. Total effect (black line) is calculated as the sum of the litter δ^13^C changes due to Suess effect (changes in atmospheric δ^13^C signature induced by fossil fuel combustion; Graven et al., 2017; blue line) and the δ^13^C changes due to CO_2_ fertilization effect and its estimated impact upon photosynthetic discrimination (red line; Feng & Epstein, 1995). The initial litter δ^13^C is calculated based on the site measurements of δ^13^C for the present day (see section 2.4) and the relation of Feng and Epstein (1995) to backwards evaluate the δ^13^C values of the litter for each year. The large increase in δ^13^C for the panel d) was due to a transition between C3 and C4 species leading to a decrease in photosynthetic discrimination.

We simulated the Suess effect on soil ^13^C by creating a time series of yearly δ^13^C litter inputs, taking into account the effects of atmospheric δ^13^C (Keeling et al., 2017) and an additional effect of atmospheric (CO_2_) variation on isotope discrimination, assuming a 2‰ depletion for each 100 ppm increase in pCO_2_ (Feng & Epstein, 1995). A customized time series was created for each site simulation, using as a reference point either δ^13^C measured in the leaves or, lacking that, in the uppermost soil layer (Figure 2).

#### 2.3.2 Difference in the Isotopic Composition of Roots and Plant Leaves/Litter

To reproduce the discrimination in isotopic composition between roots and plant leaves/litter due to post‐photosynthetic discrimination (Hobbie & Werner, 2004), we introduced a new model parameter (“enrich”) to enrich the input δ^13^C in belowground litter (assumed to be roots) compared to aboveground litter (assumed to be leaves). We additionally assumed an enrichment factor of +1‰ between belowground and aboveground matter based on averaged published data (Brugnoli & Farquhar, 2000; Schweizer et al., 1999; Werth & Kuzyakov, 2010).

#### 2.3.3 Isotopic Discrimination Associated With Heterotrophic Respiration

Microbial discrimination during litter and SOC decomposition that causes the respired CO_2_ to be depleted in ^13^C relative to the litter and SOC residues has been hypothesized (Ehleringer et al., 2000), but no clear evidence has been found. Nevertheless, most models of soil ^13^C (e.g., Ågren et al., 1996; Jagercikova et al., 2017; Poage & Feng, 2004) represent the enrichment of δ^13^C associated with the decomposition of organic matter. We represented ^13^C discrimination associated with heterotrophic respiration to test the relevance of this process. We assumed that δ^13^C discrimination occurs during heterotrophic respiration by enriching SOC ^13^C compared to CO_2_ by a discrimination factor of 0.1‰, following (Jagercikova et al. (2017). This factor is fixed with time and does not depend on the decomposition rate.

#### 2.3.4 Differential Preservation of Compounds (Lignin)

Differing decomposition rates of specific chemical compounds introduce isotopic shifts in bulk SOC, with relative lignin contents causing an isotopic shift to more depleted δ^13^C than the bulk SOC (Gleixner et al., 1993, 2005). Moreover, grassland lignins are degraded more efficiently than softwood lignins (Dignac et al., 2005). Sites with lower lignin contents (e.g., grasslands) should therefore have higher ^13^C enrichment than sites with other vegetation cover, such as forests. The difference in lignin content among sites can then be specifically parameterized by changing the lignin/carbon (L/C) ratio for each site, selecting lower ratios for grasslands or croplands as a proxy for their lower SOC lignin contents.

ORCHIDEE‐SOM does not explicitly differentiate lignin components, so introducing δ^13^C discrimination between lignin and bulk components is not straightforward. To be able to simulate a lignin‐dependent ^13^C, we would need to calculate the amount of ^13^C in above and belowground litter as a function of the L/C ratio, assuming that 1) the plant material has two isotopic compartments (lignin and nonlignin), 2) all lignin goes first to structural litter and the rest to metabolic litter, and 3) the difference in δ^13^C between lignin and bulk litter is −4‰, a typical value found in the literature (Dignac et al., 2005; Gleixner et al., 2005). We developed and coded the mass‐balance isotopic equations from this approach, but the L/C ratio had only a minimal effect on the distribution of litter between metabolic and structural pools, so the effect of the discrimination between lignin and nonlignin δ^13^C had a minor impact on the model outputs. We therefore deferred representation of differential compounds preservation in ORCHIDEE‐SOM after assessing the trade‐off between model complexity and improvement.

### 2.4 Site Descriptions

Four long‐term experimental sites with available measurements of SOC stocks and soil δ^13^C in the Parisian Basin in France were used to evaluate the model. The observed stock of ^13^C along the soil profile was first tested against soil δ^13^C measurements from two sites with different vegetation covers as follows: Feucherolles, a temperate deciduous forest and Mons, a temperate C3 grassland with no change in vegetation cover for centuries, which helps us to evaluate how the model reproduces long‐term process and changes. We also used data from two cropland sites where C4 vegetation had been planted in C3 soils, Closeaux and Boigneville, to assess the simulated shifts in δ^13^C with short‐term changes (a few decades) in vegetation cover.

The four sites have similar temperate climates, with mean annual precipitation between 600 and 700 mm and mean annual temperatures between 10 and 12 °C (Table 2). The long‐term Feucherolles and Mons experimental sites have been intensively studied (Jagercikova et al., 2014; Keyvanshokouhi et al., 2016). Both sites have Luvisol soils around 15, 000–16, 000 years old. Mons is a grassland with well‐drained soil, and Feucherolles is an oak forest where clay and gritstone deposits extend to a depth of approximately 1.5 m. The clay content is about 20% at both sites, but the pH ranges from 6 in Feucherolles to 7 in Mons (Table 2; Jagercikova et al., 2014). The soil profiles at Feucherolles and Mons were sampled in 2011, and the samples were analyzed for carbon stock, δ^13^C isotopic ratios and other soil properties (Jagercikova et al., 2017). ^13^C/^12^C ratios were analyzed using the online continuous elementary analyzer coupled with an isotopic ratio mass spectrometer (Finigan Delta+XP, Bremen Germany). The results are expressed in δ^13^C per mil (‰) against the international standard V‐PDB.

**Table 2:** Site Characteristics

Coordinate	Feucherolles	Mons	Closeaux	Boigneville
	48.9°N, 1.97°E	49.87°N, 3.03°E	48.8°N, 2.08°E	48.32°N, 2.37°E
Land use	Oak forest	Grassland	Wheat cultivation Wheat‐corn rotation	Wheat cultivation Wheat‐corn rotation
Mean Annual Precipitation (mm)	660	680	680	630
Mean Annual Temperature (°C)	11.2	11	11	10.4
Soil type	Luvisol	Luvisol	Cambisol, Fluvisol	Luvisol
Soil pH	5.9	6.89	6.8	6.8
Soil bulk density (g cm−^3^)	1.34	1.4	1.5	1.4
Soil clay fraction (%)	20	23	17.4	22

Boigneville is an experimental site, also with Luvisol soil (Table 2; Jagercikova et al., 2014), that was used for an experiment of C3‐C4 succession. The soil pH is 6.8 and the clay content 22% (Table 2). The experiment contained various rotations from 1970 that compared continuous wheat (C3) and continuous maize (C4) crop cover. Wheat and maize plots were sampled in 1985 and 1987, respectively. More details on the sampling and analytical methods have been provided by Balesdent et al. (1990). We used δ^13^C isotopic ratios measured from untilled plots. The soil at the Closeaux site is classified as a Eutric Cambisol. The mean clay content of the soil measured in 2003 was 17.4%, and the mean pH was neutral (Table 2). The experiment on C3‐C4 succession at Closeaux was carried out by planting maize in 1993 in a wheat field that had never previously grown any C4 plants. The plots were managed following standard agricultural practices for similar field crops in the Parisian Basin (Rasse et al., 2006). The experimental plots (maize and control plots) were first sampled in 2003 and again in 2016, and the soil samples were analyzed for carbon stock, δ^13^C isotopic ratios, and other soil properties (Rasse et al., 2006).

### 2.5 Simulation Setup

ORCHIDEE needs the following environmental drivers as forcings as follows: air temperature, wind speed, solar radiation, air humidity, precipitation, and atmospheric CO_2_ concentration. We extracted the meteorological forcings for each site from the Climatic Research Unit National Centers for Environmental Prediction (CRUNCEP) data set with a resolution of 0.5°, which is a combination of two existing data sets as follows: the CRU TS.3.2 0.5 × 0.5° monthly climatology (Mitchell et al., 2004) and the NCEP reanalysis (Kalnay et al., 1996).

For sites and regional simulations over Europe, we followed similar protocols. Before running over a historical period (1901–2011), we ran the model for approximately 13,000 years by looping over the meteorological data for the available period for each site until all soil variables reached a steady state (spin‐up). We considered that the model reached equilibrium when the pool increase was less than 1‰ year^−1^ during the last decade of the spin‐up. The meteorological forcings were randomly selected between 1901 and 1910 during the spin‐up, and the atmospheric CO_2_ concentration was maintained at 296.13 ppm, representing 1901 (Keeling & Whorf, 2006). For the spin‐up, litter δ^13^C was fixed to a specific value calculated for 1901, taking into account the ^13^C depletion due to the Suess effect and CO_2_ fertilization (see section 2.2.1, Fig. 2) and using as a reference the best approximation for litter δ^13^C presently available for each site. For European simulations we followed the same approach but we used δ^13^C values of litter collected literature mainly gathered by Diefendorf et al. (2010) that we completed with data from Schweizer et al. (1999). For Closeaux and Boigneville, we used the δ^13^C of wheat leaves measured in 2005 as a reference. δ^13^C had not been measured for leaves or litter at Feucherolles or Mons, so we used δ^13^C measured in the uppermost soil layer as a reference for calculating the input δ^13^C for 1901 (Table 2). Site‐specific measurements were used for pH, clay content, and bulk density. For the European scale simulation we used pH, clay content, and bulk density from the harmonized world soil database (HWSD; Nachtergaele et al., 2012) product.

The state of the ecosystem at the last time step of the spin‐up was then used as the initial state for running the historical simulations with outputs written at time steps of 1 year for 1901–2011 for Feucherolles, Mons, Boigneville, and Europe and 1901–2016 for Closeaux. The historical simulations were driven by 6‐hourly meteorological forcings and yearly time series of CO_2_ and litter δ^13^C data (Figure 2). We performed two historical simulations for Closeaux and Boigneville, a “control” simulation, with a yearly variation of litter δ^13^C based on the Suess effect and CO_2_ fertilization, and a “C4” simulation, where an abrupt change in litter δ^13^C to −12.8‰ was simulated for the year of the maize crop. Finally, it is important to note that for parameters related to bulk soil carbon, we kept the original parameters values published by Camino‐Serrano et al. (2018).

### 2.6 Model Experiments

We performed two sets of simulations after the introduction of ^13^C and its related processes into ORCHIDEE‐SOM as follows:

1. First, we evaluated the full‐model performance by simulating the soil δ^13^C profiles at the four sites (Feucherolles, Mons, Closeaux, and Boigneville). For these simulations, we used the complete model with the three processes of ^13^C discrimination “activated,” that is, the Suess effect and CO_2_ fertilization, root enrichment and discrimination.
2. Second, we tested the relative importance of each of the processes of ^13^C discrimination included in the model by activating one at a time and comparing the model outputs. These simulations were performed only for Feucherolles and Mons, the two sites where the abundance of ^13^C had been measured, thus discarding the additional short‐term effect of changes in vegetation cover at the Closeaux and Boigneville experimental sites.
3. Finally, we ran a simulation over Europe with the full model including the three processes of ^13^C discrimination (Suess effect and CO_2_ fertilization, root enrichment, and discrimination) for the period 1901–2011.

The full set of simulations is shown in Table 3.

**Table 3:** Set of Simulations Performed. Crosses Indicate the Processes That Have Been Included in Each Simulation

Location	Set 1: Full‐model simulations		
	Suess effect	Root enrichment	Microbial Discrimination
Feucherolles	X	X	X
Mons	X	X	X
Closeaux_C4	X	X	X
Closeaux_control	X	X	X
Boigneville_C4	X	X	X
Boigneville_control	X	X	X
Europe	X	X	X
	Set 2: Relative importance of processes of ^13^C Discrimination		
Feu_Suess	X		
Feu_Enrich	X	X	
Mons_Suess	X		
Mons_Enrich	X	X	

### 2.7 Comparison of Model Results With Measured Data

At the European scale, we compared the SOC stocks (kg m^−2^) with those given by the HWSD v1.2 regridded at 2° to match with the resolution of the model. The heterotrophic respiration was compared with the global products of (Hashimoto et al., 2015) who used a climate‐driven model of soil respiration based on a global soil respiration data set to predict the heterotrophic fluxes at monthly time step during the 1901–2012 period. At the site level, SOC stocks (kg m^−2^), and soil δ^13^C measured along the soil profiles at the four sites were compared with the soil δ^13^C simulated with ORCHIDEE‐SOM. The simulated and measured soil δ^13^C were compared using the mean squared deviation (MSD) and its three components: the squared bias (SB), the non‐unity slope (NU) and the lack of correlation (LC; Hugh et al., 2003). SB is an indicator of the mean bias of the simulation from the measurement, NU provides information on the ability of the model to reproduce the magnitude of fluctuations among the measurements, and LC indicates the ability of the model to reproduce the shape of the data. The lower the MSD, SB, NU, and LC, the better the fit. These indicators have been shown to be useful for comparing model‐observation fits among models (e.g., Guenet et al., 2013).

The four statistical indices were calculated for 10 site simulations (Table 3) to identify the simulation with the best fit to the measured δ^13^C (lowest MSD) in the following cases: 1) for Feucherolles and Mons, we compared the model with different numbers of processes included, and 2) for Closeaux and Boigneville, we compared the “control” and “C4” simulations. The intervals of soil depth of the model outputs and the measurements were homogenized by interpolating the data to common depth intervals defined for each site. The simulations and data were then compared for each depth interval, so the statistical indices indicated the performance of the model for identifying spatial patterns (soil profiles). The MSDs of the simulations for Feucherolles and Mons were directly comparable because the measurements were the same, so the MSDs for the Closeaux and Boigneville simulations were based on different field measurements (control and C4 plots).

## 3 Results

### 3.1 Vertical Profiles of SOC Stocks

**Figure 3:**
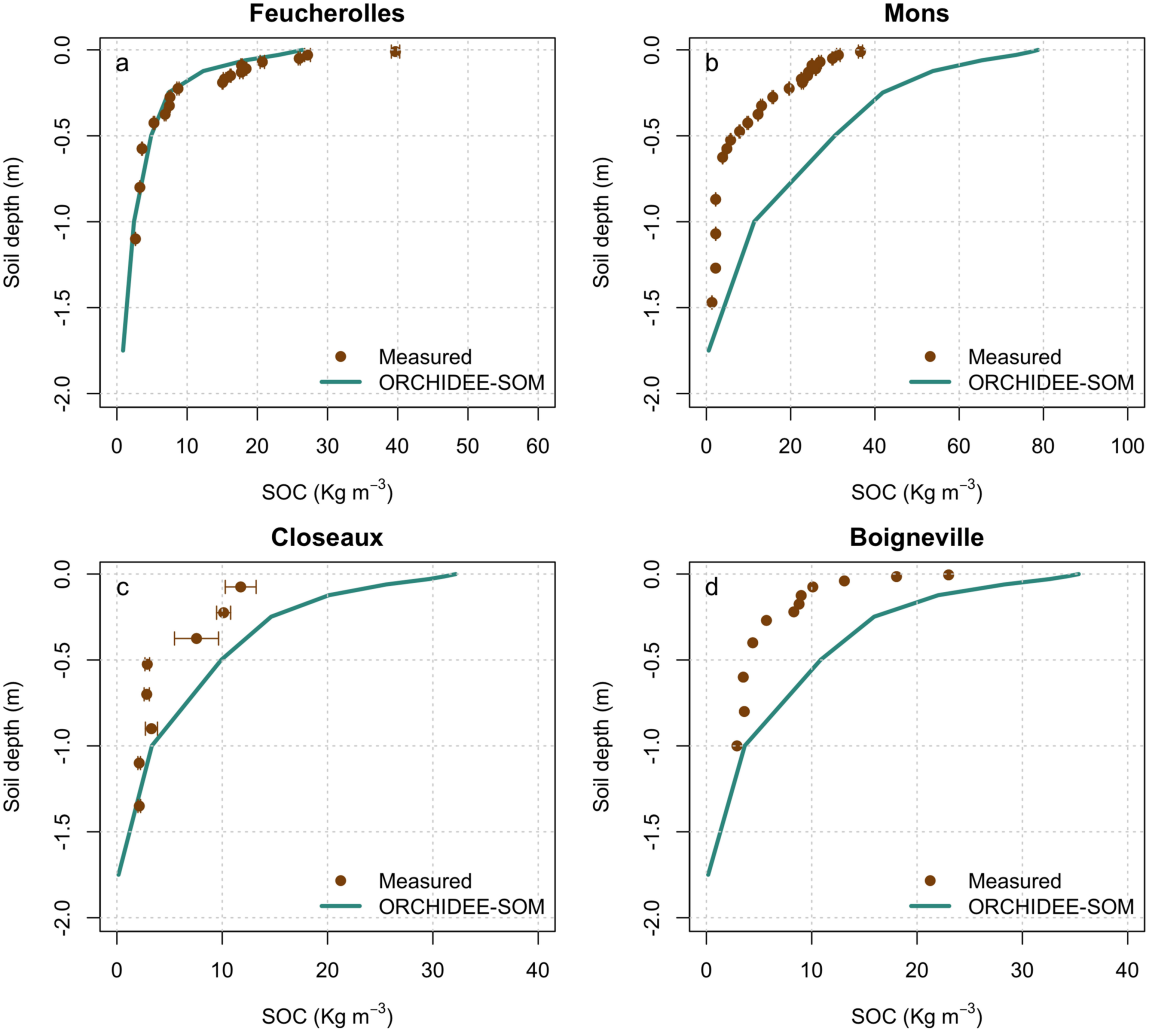
Simulated (green) versus observed (brown) soil organic carbon (SOC) stocks for (a) Feucherolles, (b) Mons, (c) Closeaux (control plot), and (d) Boigneville (control plot). The error bars represent the standard deviations of the observed SOC stocks (standard deviations were not available for Boigneville).

The model simulations indicated that the shape of the vertical SOC profiles was well reproduced by ORCHIDEE‐SOM, but the total SOC stock was often overestimated, particularly for the upper soil layers, except for SOC in the Feucherolles deciduous forest (Table 4, Figure 3). The modeled vertical profile of SOC at Feucherolles was simulated very well (Figure 3a). The model, however, was not able to accurately simulate the high SOC measured near the surface there (1‐cm depth), which may account for the 17% underestimation of the total SOC stock compared to the measured total SOC calculated to a depth of 1 m (Table 4). In contrast, simulated total SOC stock to a depth of 1 m was overestimated by more than 200% at the Mons grassland site (Table 4). Total SOC stocks for Closeaux and Boigneville were also overestimated by the model, but more moderately (by 24 and 63%, respectively). The overestimation of the modeled SOC in the upper soil layers for the Closeaux site was expected, because the current soil module in ORCHIDEE‐SOM does not yet account for the effect of plowing, so the concomitant reduction in SOC stocks cannot be reproduced. Nevertheless, Boigneville has been unplowed for some years, and model overestimation was due to other model errors such as root profile or net primary production.

**Table 4:** Measured (±SD) and Simulated SOC Stocks Calculated to a Depth of 1 m

Location	Measured SOC (kg C m^−2^, *Mean±SD*)	Modeled SOC (kg C m^−2^, *Mean±SD*)	Difference (%)
Feucherolles	8.4 ± 0.08	6.9	−17
Mons	11.0 ± 0.1	33.7	206
Closeaux	9.5 ± 0.9	11.8	24
Boigneville	8.3	13.4	63
Europe	24.1	11.0	54

*Note*. European data was taken from HWSD.

Abbreviation: SOC, soil organic carbon

### 3.2 Vertical Profiles of the Stock of ^13^C: Mons and Feucherolles

**Figure 4:**
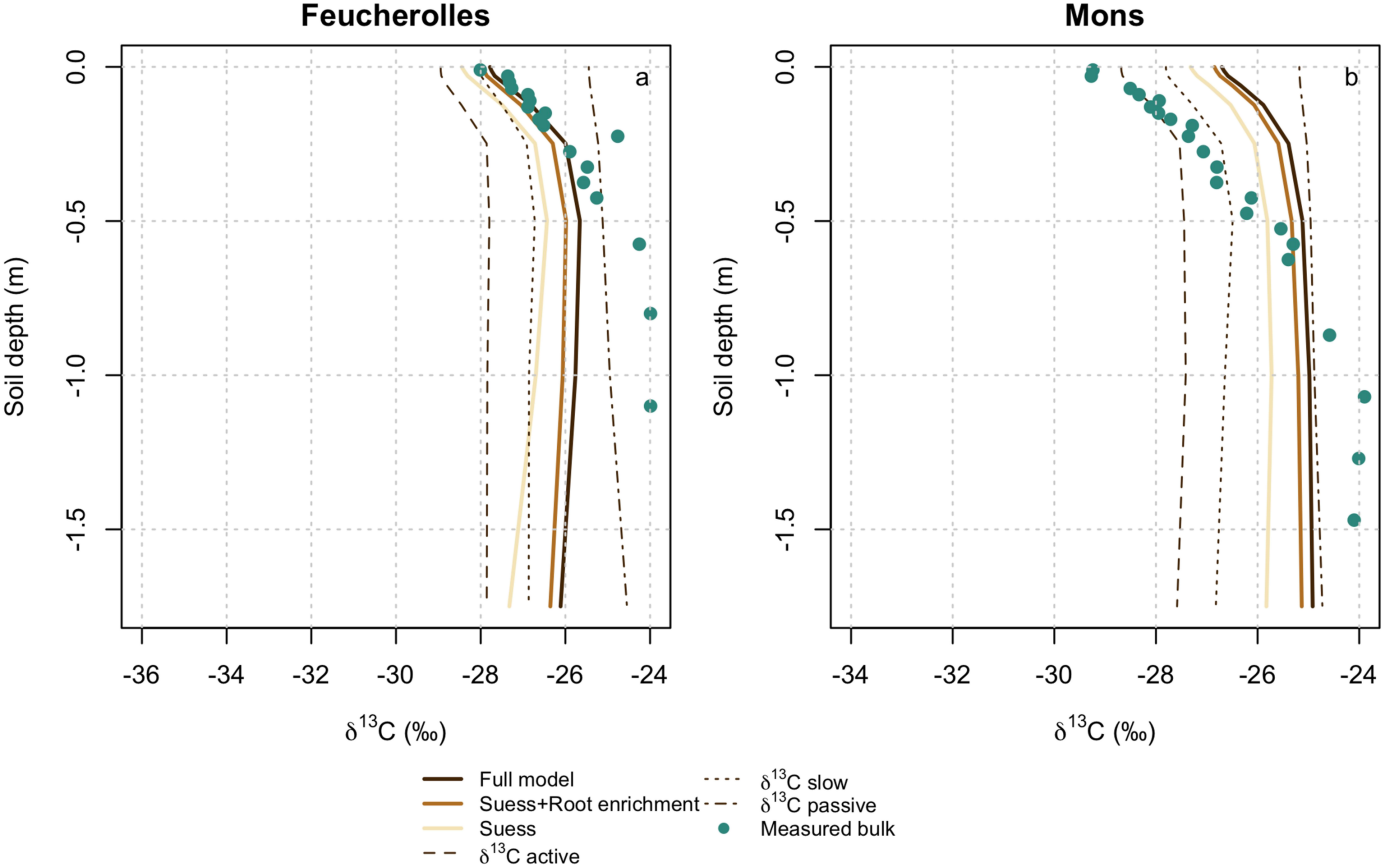
Comparison between simulated and measured soil δ^13^C profiles for the two sites used to model the observed stock of δ^13^C as follows: (a) Feucherolles and (b) Mons. Three model versions are represented the full model (Suess effect, CO_2_ fertlization effect + root enrichment + microbial discrimination), the model representing the Suess effect, CO_2_ fertilization effect, and root enrichment, and the model representing only the *Suess* effect, CO_2_ fertlization effect. δ^13^C simulated by the full model has been decomposed into δ^13^C of the active, slow, and passive pools. The standard deviations from the observations were too small and are obscured by mean values.

**Figure 5:**
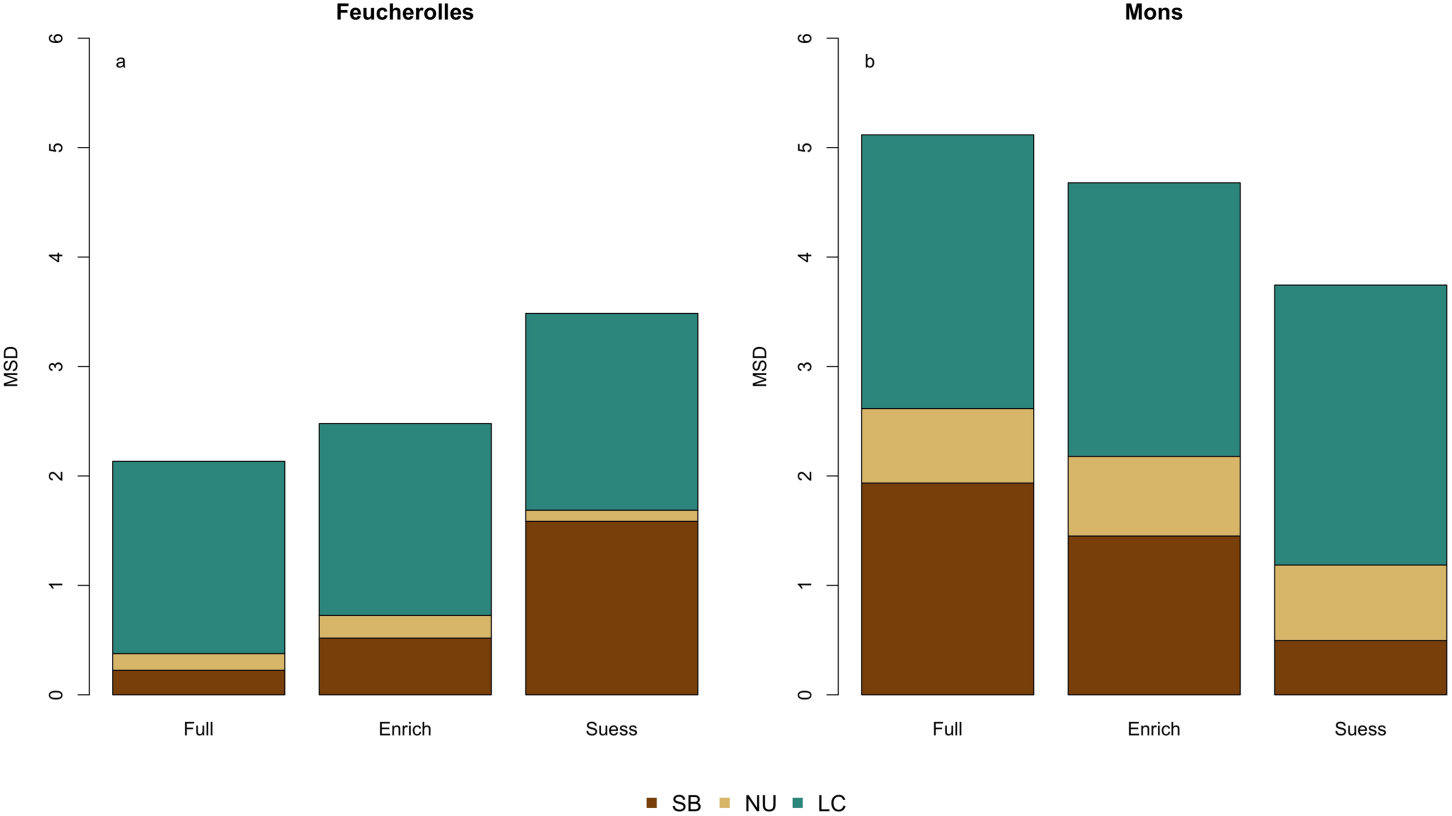
Components of mean squared deviation (MSD) calculated for δ^13^C for the three model formulations in Figure 4 for (a) Feucherolles and (b) Mons. The three components are lack of correlation (LC), non‐unity slope (NU) and squared bias (SB), while the MSD is the sum of these components. The lower the MSD, the better the fit.

The shape of the δ^13^C profile was well reproduced by the model for Feucherolles when taking only the natural variations in δ^13^C into account, but δ^13^C was overestimated along the full soil profile (Figure 4a). The simulated δ^13^C for Mons was overestimated in the top soil layers but underestimated in the deepest soil layers (Figure 4b). We ran three model simulations for Feucherolles and Mons as follows: 1) the full model (Suess effect and CO_2_ fertilization, root enrichment, and discrimination), 2) the model with the *Suess* effect and CO_2_ fertilization, and root enrichment and 3) the model representing only the Suess effect and CO_2_ fertilization. The results from the three simulations were compared visually (Figure 4) and statistically by calculating the MSD components (Figure 5). The variations in δ^13^C produced by adding or removing the processes of ^13^C discrimination were generally very small compared to the observed variation in δ^13^C identified by the measurements. The simulated δ^13^C over the vertical profile varied by roughly 2‰ with depth, whereas the observed δ^13^C varied by roughly 4‰, which is higher than the averaged difference between the different simulation setup. That is, the observed ^13^C variability was higher than the variation of δ^13^C that the current version of ORCHIDEE‐SOM was able to simulate.

For Feucherolles the “full model” gave the best model‐data fit (MSD = 2.1; Figures 5a and 5b, respectively). The model improvements were due to a reduction in the standard bias of the simulations from the measurements (lower SB), that is, due to a reduction in the “gap between magnitudes,” but the ability of the model to reproduce the fluctuation among the measurements (indicated by NU) or the shape of the data, that is, the vertical profile (indicated by LC), remained almost constant (Figure 5). For Mons, the MSD value for the full model was relatively small (MSD = 5.1) but better performances were observed when only the Suess effect and CO_2_ fertilization was taken into account through a reduction of the standard bias (lower SB).

### 3.3 Vertical Profiles of ^13^C in Scenarios of Changes in C3/C4 Vegetation

**Figure 6:**
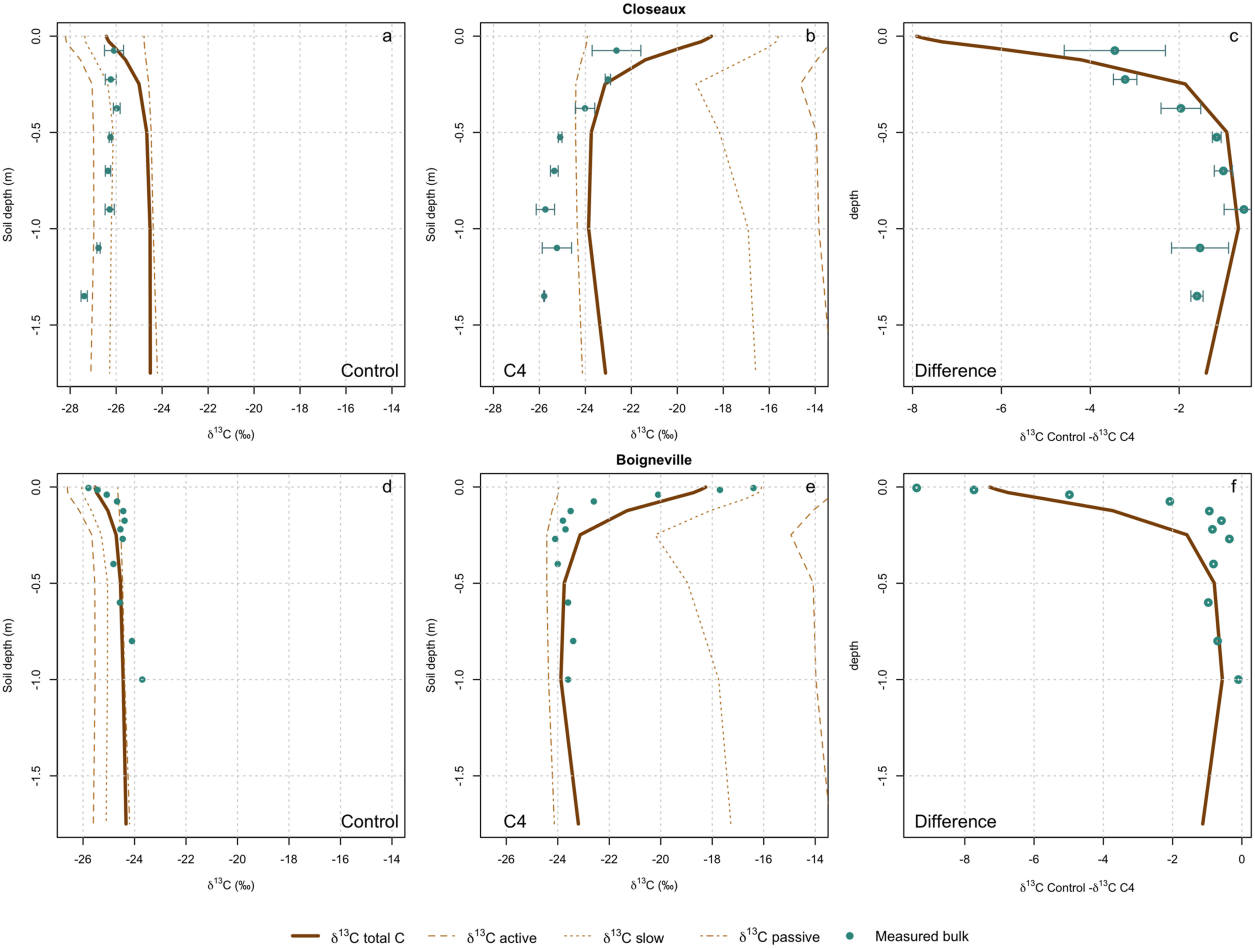
Comparison between simulated and measured soil δ^13^C profiles for the two sites with C3/C4 rotation: Closeaux (a, b, and c) and Boigneville (d, e, and f). The comparisons are for the results from the control plot (under C3 vegetation), the results from the C4 plot and the difference between δ^13^C in the control and C4 plots. The simulated δ^13^C has been decomposed into δ^13^C of the active, slow, and passive pools. Errors bars from data are the observed standard deviation. Please note that for Boigneville only one core was sampled, and consequently we could not calculate the standard deviation.

**Figure 7:**
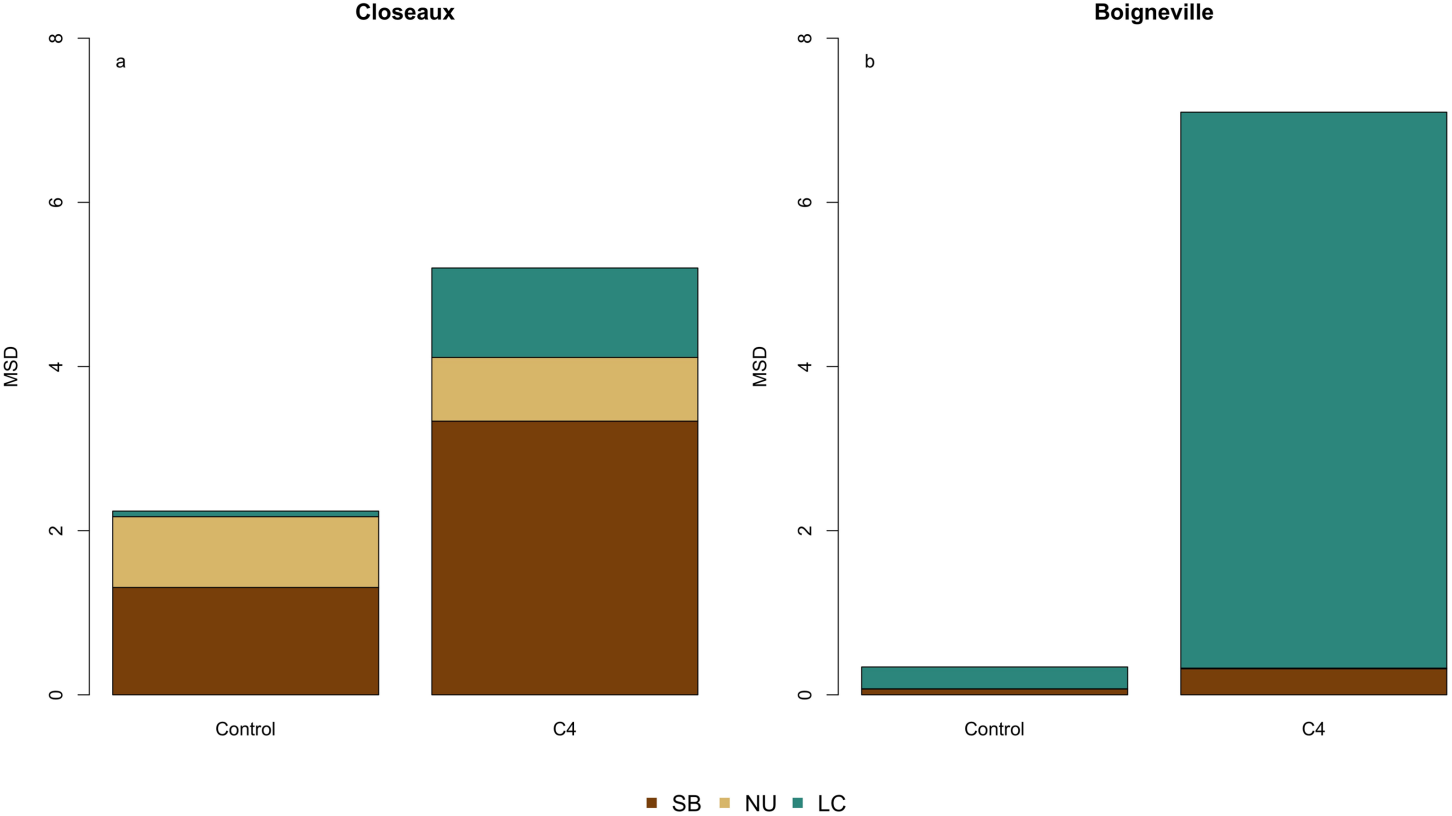
Components of mean squared deviation (MSD) for the model formulations for (a) Closeaux and (b) Boigneville. The three components are lack of correlation (LC), non‐unity slope (NU), and squared bias (SB), while the MSD is the sum of these components. The lower the MSD, the better the fit.

The model was able to identify the change in the isotopic signature after the shift from C3 to C4 plants at the two croplands sites (Figure 6). More specifically, the model matched the observations well in the topsoil but missed the slight depletion in δ^13^C in the deeper soil layers at Closeaux, for both the control and the C4 plot (Figures 6a and 6b), which can also be seen by the values of the MSD components (Figure 7a). The model satisfactorily reproduced the shift in the vertical δ^13^C profile toward ^13^C enrichment in surface soil when the change to C4 plants was simulated. LC, however, increased slightly in the C4 simulation, indicating a worsening of the simulated shape of the vertical profile. The shape of the simulated vertical profile of δ^13^C differed from the observed profile because the model simulated a slight, unobserved, ^13^C enrichment at the bottom of the profile (Figure 6b).

The model‐data fit was very satisfactory for the control plot at Boigneville, capturing both the shape and magnitude of δ^13^C (Figure 6d), indicated by its very low MSD (0.34; Figure 7b). The model reproduced the shift in the vertical profile of δ^13^C when the C4 plot was simulated, even though LC increased in this simulation. As with the Closeaux site, this indicated a poorer simulation of the vertical profile. The model also simulated a slight ^13^C enrichment at the bottom of the profile that was not observed. Finally, ORCHIDEE‐SOM reproduced the differences between the control and C4 δ^13^C at the two experimental sites (Figures 6c and 6f). The differences between δ^13^C of the control and C4 plots at each soil depth indicated that the “background effect” of the long‐term changes in ^13^C could be removed, isolating only the effects of the change in C3/C4 vegetation.

### 3.4 SOC Stocks, δ^13^C, and Heterotrophic Respiration at European Scale

**Figure 8:**
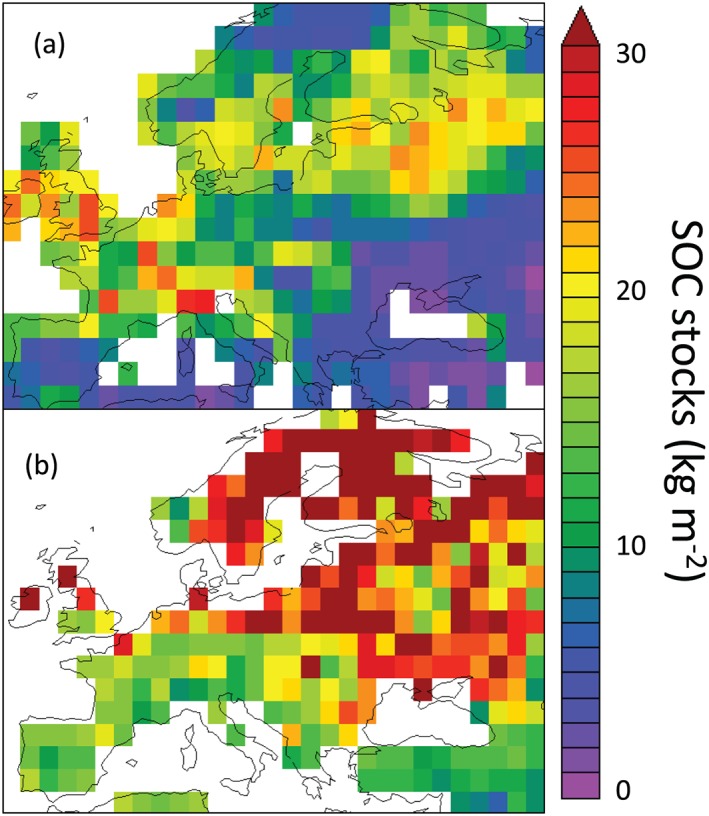
SOC stocks (kg C m^−2^) at 1‐m depth over Europe (a) simulated by ORCHIDEE‐SOM and (b) estimated by HWSD. SOC, soil organic carbon.

**Figure 9:**
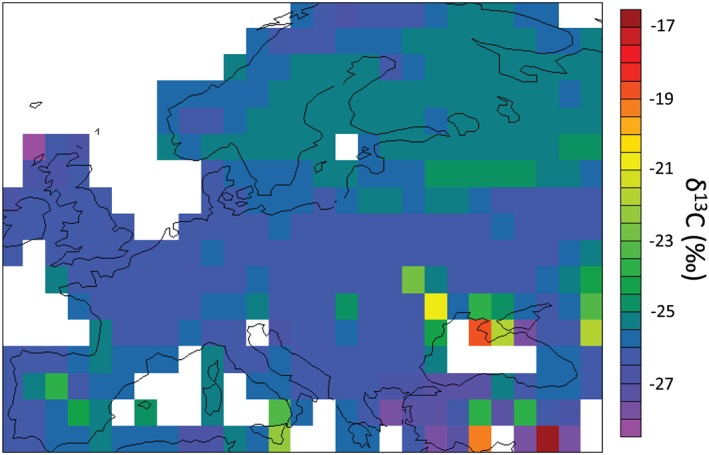
Map of soil δ^13^C values over Europe from ORCHIDEE‐SOM (average over the period 2001‐2011) for simulations with the three processes of ^13^C discrimination activated (*Suess* effect and CO_2_ fertilization, root enrichment and discrimination).

At the European scale, the model underestimated total SOC stocks at 1 m by 54% (Table 4), although this bias was not equally distributed over Europe (Figure 8), and it was not able to reproduce high SOC stocks HWSD data for Northern Europe. Nevertheless, the model provides reasonable results for Western Europe. The δ^13^C predicted by the model ranged between −28.5‰ and −17.5‰ (Figure 9), with its spatial variability mainly due to land cover and in particular, the proportion of C4 plants in a given pixel. Model average δ^13^C was −26‰, consistent with most local measurements published in the literature over this region (see e.g., Balesdent et al., 1993; Fontaine et al., 2007; Guenet et al., 2012).

**Figure 10:**
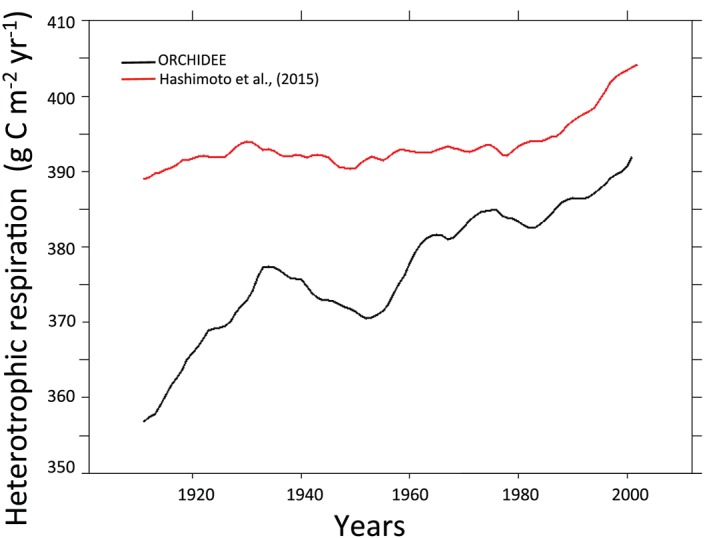
Twenty‐year running mean of the heterotrophic respiration over Europe calculated by ORCHIDEE‐SOM (black line) and estimated by Hashimoto et al. (2015; red line).

The model slightly underestimated average heterotrophic respiration over Europe, which nonetheless increased over the study period in both observations Hashimoto et al. (2015) and ORCHIDEE‐SOM output. On the other hand, the simulated rate increase calculated by ORCHIDEE‐SOM was higher than that proposed by Hashimoto et al. (2015; Figure 10). Thus, while observation‐based respiration rates were relatively stable over the 20th century (~390 g C m^−2^) Hashimoto et al. (2015) and increased from the 90s to 405 g C m^−2^, the model predicted fluxes of 355 g C m^−2^ at the beginning of the simulated period, reaching roughly 390 g C m^−2^ at its end.

## 4 Discussion

We applied a set of modifications to the original ORCHIDEE‐SOM soil module to simulate ^13^C stocks and the processes leading to ^13^C discrimination along the soil profile. These processes were 1) the Suess effect and CO_2_ fertilization effect, 2) ^13^C enrichment of root material compared to leaves, and 3) ^13^C discrimination associated with heterotrophic respiration. Furthermore, it is important to note that the ^13^C entering the soil is not directly calculated by the plant module but instead is imposed directly on the litter pool. To our knowledge, only one global model, the CLM4.5, represents the stable carbon isotopic tracer in the soil, but unlike the ORCHIDEE‐SOM soil module presented in this study, CLM4.5 does not include any representation of post‐photosynthetic ^13^C discrimination (Duarte et al., 2017; Raczka et al., 2016). The version presented here is therefore the first soil module that can model the transport of soil ^13^C through the soil column and the post‐photosynthetic ^13^C discrimination at large scales. We discuss the implications of the results presented above in the following section.

### 4.1 SOC Stocks

Our model generally overestimated SOC stocks, except for the simulation for the Feucherolles deciduous forest (Figure 3). The mismatch between measured and modeled SOC may arise from biases in simulated net primary production (NPP) or to incorrect parameters given for SOC turnover times. Looking at the first of these two causal factors, turn to the Mons site, which is the only one for which primary production data exist. For this site, the French Technical Institute for Pasture estimated NPP of 6.7 t·ha^−1^·year^−1^ based on annual yields, versus model predictions of 7.5 t·ha^−1^·year^−1^. Thus overestimation of NPP by the model can at least partially explain the total SOC overestimation for this site. For the other sites, we do not have enough information to identify the cause of the general overestimation of SOC. ORCHIDEE‐SOM can nevertheless successfully simulate SOC stocks when NPP is constrained (Camino‐Serrano et al., 2018), indicating that the overestimation of the simulated SOC in our study may be due to a bias in the simulated aboveground production. Nevertheless, when applied at the European scale, mismatch in NPP may not be the only explanation for the mismatch in SOC stock. Indeed, most of the mismatches are located in Northern Europe where peatlands are a significant contributor to the total SOC stock. This version of the model does not yet include some recent developments done to include peatland in ORCHIDEE (Qiu et al., 2018), which likely explains the underestimation of the SOC stocks over Northern Europe.

Turning to the second causal factor, because δ^13^C measurements can be used as an observational constraint for improving SOC turnover times (Acton et al., 2013; Luo et al., 2016; Wang et al., 2018) development and evaluation of the soil ^13^C module in ORCHIDEE‐SOM may help, in the future, to better constrain the SOC turnover times and hence simulated SOC stocks at broad spatial and temporal scales (Balesdent et al., 2018a, 2018b; Lawrence et al., 2019), whose output we discuss below.

### 4.2 Observed Vertical Profiles of δ^13^C: Relative Importance of the Different Processes Involved

We first evaluated the ability of the model to reproduce the observed stock of ^13^C at two sites that have continuously had C3 plants growing on the soil. At these sites, the observed vertical profile of δ^13^C was expected to be due to the Suess effect and other natural processes. We found that the “full” version of the model (representing the Suess effect and CO_2_ fertilization, root ^13^C enrichment, and the ^13^C discrimination of heterotrophic respiration) was not always the best for matching observations. For instance, on the one hand, the Feucherolles simulations improved when we applied the full model to that site, whereas for Mons the full model tended to overestimate the average δ^13^C along the profile. It is important to note that average δ^13^C along the profile is quite sensitive to the initial δ^13^C value for the litter (supporting information, Figure S1). Deep SOC was clearly enriched in ^13^C at the Mons grassland site, and the Suess effect and CO_2_ fertilization may account for only about 50% of the ^13^C enrichment with soil depth. Thus, processes not represented in the model such as the preferential decomposition of some components might explain the mismatch between model output and observation (Högberg et al., 2005; Wynn et al., 2006).

Accounting for all processes included in the full model therefore unsurprisingly improved the simulated vertical profile of δ^13^C for those sites. Our model represents ^13^C discrimination and root enrichment relative to leaves that can lead to an enrichment of deep soil δ^13^C, but the modeled ^13^C enrichment occurs along the entire soil profile and not only in the deep layers, contrary to what we would expect regarding this modelling approach. The model‐data fit consequently improved using the full model; however, it remains a long way from adequately representing reality with regard to ^13^C enrichment in deep soils.

The input litter δ^13^C selected for the site simulations was key for reproducing the correct δ^13^C profile at site level. In fact, simulated profiles were highly dependent on the choice of input δ^13^C, indicated by the results of a small sensitivity analysis (Figure S1). For example, the model‐data fit for the vertical profile of δ^13^C for Feucherolles would have improved considerably had we selected an input litter δ^13^C of −28‰, moving simulated profiles to the left (Figures 4a and S1). We selected the best information for litter δ^13^C that was available at the time of writing, based on measurements of soil or leaves (Table 2), and we derived the values for each year based on the Suess effect and CO_2_ fertilization. Litter δ^13^C was unfortunately not measured at all sites. In Closeaux and in Boigneville litter δ^13^C was measured in 2005 and in 1987, respectively, with values of leaves and straw for the C3 plots (resp. C4) of −27.9‰ (resp. −12.1‰) for Closeaux and −26.3‰ (resp. −12.5‰) for Boigneville. Our estimation gave values of −28.4‰ (resp. −13.2‰) and −27.1‰ (resp. −13.3‰) for Closeaux and Boigneville for the corresponding years. We thus recommend that a fully climate coupled photosynthetic discrimination model be included in future experiments and have confidence that the modeled δ^13^C profile in ORCHIDEE‐SOM will improve when our soil module is merged with the representation of ^13^C in vegetation, since this will give us more realistic litter δ^13^C values.

### 4.3 Vertical Profiles of δ^13^C Under C3/C4 Vegetation Changes: Long‐term Versus Short‐term ^13^C Variations

Transitions of vegetation from C3 to C4 photosynthesis (or vice versa) or species shifts that change the input δ^13^C to litter are recorded in the SOC δ^13^C. The model more accurately reproduced ^13^C profiles due to vegetation changes at Closeaux and Boigneville than background ^13^C values which arise from site history (Figure 6). Modeled δ^13^C differences between the control and C4 plots matched observed (control‐C4) differences when the background ^13^C signature was subtracted (Figures 6c and 6f), indicating that the physical mixing of SOC derived from C3 and C4 vegetation in the short term was represented by ORCHIDEE‐SOM. The physical mixing of two carbon sources with different ^13^C signatures during the first decades occurred primarily in the upper soil layers, where δ^13^C of the total SOC pool is mainly driven by the ^13^C of the active SOC pool. This pool decomposes in the short‐term (1 year), suggesting that the model accurately represented the relative proportion of the turnover times of the various SOC pools in the upper soil layers, where SOC production from biomass residues is highest (Wynn et al., 2006).

However, the model failed to simulate δ^13^C measured in deep soils. Deep soil ^13^C signatures represent long‐term processes as follows: 1) the long‐term history of the land cover that represents the physical mixing of bulk SOC derived from biomasses with different isotopic signatures and 2) SOC processing and stabilization. Stabilization processes in deep soils are related to interactions with the mineral matrix, physical protection, and fresh organic matter limitations for the decomposers (Fontaine et al., 2007; Wordell‐Dietrich et al., 2017). The long‐term history of land cover, and a lack thereof, is an important factor limiting accurate reproduction of vertical profiles of δ^13^C in our model. The isotopic signature of deep soil (>1 m) may represent paleoecological changes of the last millennia (Cerling et al., 1989). Detailed information about the land‐cover history at the site level that extends back a thousand years, however, is not available at the scales needed to run a global model such as ORCHIDEE‐SOM. Land‐cover history can sometimes be more important than the observed variation due to the internal processes of ^13^C discrimination. For example, the large ^13^C enrichment in the deep soil of Mons (the grassland site) may have been due to past vegetation cover that we were unable to model. Simulating the long‐term land‐cover history in global carbon models, which are normally spun‐up for a thousand years under constant conditions, that is, fixing the input parameters like litter δ^13^C, would present serious computational challenges even if the information was available.

Similarly, the failure to satisfactorily model the vertical profile of total δ^13^C may indicate that the relative proportions of the SOC pools along the profile were not correctly simulated. In our model, ^13^C discrimination associated with heterotrophic respiration was represented by applying the same discrimination coefficient for all soil carbon pools. The modeled δ^13^C of total SOC consequently primarily represented changes in the proportional contributions of each pool to total SOC along the profile (Baisden et al., 2002). The model simulated a low proportion of passive pools in the deepest layer for all sites except Mons (supporting information, Figure S2), which could account for the simulated ^13^C depletion in the deep soil for Feucherolles, where the isotopic signal of the younger, less processed SOC pools (i.e., active and slow) was higher than expected. The small size of passive pools in deep layers, together with the low turnover times of SOC compared to the measured mean ages of SOC (He et al., 2016), led to an underestimation of the mean soil age by ORCHIDEE‐SOM. He et al. (2016) optimized the turnover times and transfer coefficients of the slowest SOC pool using radiocarbon data to correct the bias for the modeled mean age of soil carbon. We propose that this newly developed module of ^13^C may help us to better parameterize the vertically discretized model of SOC and DOC that has recently been developed and where the new parameters remain highly uncertain (Camino‐Serrano et al., 2018).

### 4.4 Model Assumptions and Future Directions

The ^13^C soil module presented here is in the early stages of development and its coupling with a plant module fully representing photosynthetic discrimination would be necessary in the future. The processes involved in the discrimination of soil ^13^C were modeled here with a simplified representation of the mechanisms involved as a first attempt to include them in a global dynamic vegetation model. The ^13^C enrichment of roots and ^13^C microbial discrimination are currently represented by adding a fixed parameter, based on published values, but this parameter does not account for any environmental factors or interactions that will change model outputs based on site‐specific conditions. For example, the level of isotopic discrimination has previously been associated with organo‐mineral associations (Baisden et al., 2002), so previous modeling attributed different discrimination factors to the transfers between SOC pools depending on their chemical recalcitrance (Baisden et al., 2002). We assumed the same discrimination factor for all SOC pools in our model. In fact, the mechanisms and factors behind ^13^C discrimination associated with heterotrophic respiration remain unresolved. Further, trying to quantify ^13^C discrimination at a high level of detail may unnecessarily increase model uncertainty, given the purpose of a large‐scale model such as ORCHIDEE‐SOM. Further research to elucidate the mechanisms behind the apparent ^13^C discrimination is needed before model parameterization at the global scale can be refined.

Other factors that account for differences in the vertical profile of ^13^C have been proposed. ^13^C enrichment with soil depth can increase within fine soil particles (Bird et al., 2003; Brüggemann et al., 2011), probably due to the preferential use by microbes of ^13^C‐enriched SOC stabilized by association with fine particles. In the model, the influence of texture on SOC decomposition is indirectly taken into account in the model by a clay modifier that decreases decomposition of the active pool with increasing clay content. Litter quality and temperature have also been proposed as factors influencing isotopic discrimination during SOC decomposition (Garten et al., 2000)., ORCHIDEE‐SOM has not yet been modified to specifically account for these environmental controls on the discrimination of ^13^C with soil depth.

Not all processes determining the isotopic signatures of soil carbon pools are represented in our model (Figure 1). Anaplerotic dark CO_2_ fixation associated with heterotrophic metabolisms, which incorporates soil CO_2_ carbon atoms into microbial biomass, may also contribute to microbial isotopic signature, but the process has yet to be quantified in situ. Diffusional discrimination is not simulated because ORCHIDEE‐SOM does not represent CO_2_ dissolved in the soil solution. The use of carbohydrates stored in plant material (represented as “starch” in Figure 1) are controlled by supply and demand and may also influence SOC isotopic signatures, but ORCHIDEE‐SOM represents carbon as three reactivity pools that are independent from the chemical constituents of that carbon, meaning we cannot model ^13^C discrimination at this level of process resolution. Finally, plant respiratory discrimination is also currently neglected but could be included when the representation of ^13^C in vegetation is merged with our model. Photosynthetic assimilation of CO_2_ derived from soil respiration, which is relevant in closed canopy ecosystems, may be added as well during integration of carbon isotopes in vegetation. We can add more processes and thus model complexity as we continue to formulate and parameterize new soil modules to reduce uncertainty, increasing the accuracy of the newly developed module.

Finally, ORCHIDEE‐SOM is a model intended for global simulations, although we applied it in this study at site and European‐scale. The extrapolation of our results is, however, limited because the model has only been tested for a temperate climate and for loamy soils with slightly acidic‐neutral pH. More model validation exercises should be carried out in different ecosystems and climates to fully evaluate model capability before global scale application. Moreover, more effort should be dedicated towards improved model parameterization and optimization procedures which often need large and detailed data sets (MacBean et al., 2016). For SOC‐related parameters, we would need full profiles of total SOC and their δ^13^C associated with all the necessary boundary conditions, which are often not available (e.g., Desjardins et al., 2006; Navarrete et al., 2016). We therefore encourage the creation of large datasets covering SOC and their associated isotopes. An optimization process may also benefit from the implementations of other tracers like ^14^C (Menichetti et al., 2016) or Cesium‐137 (Evrard et al., 2010). Another version of ORCHIDEE‐SOM has already implemented a ^14^C tracer (Tifafi et al., 2018), and using both, ^14^C and ^13^C may be useful for evaluating the model's reproduction of observed emerging properties, such as the age of primed organic carbon at different depths.

## 5 Conclusions

This study has presented modifications made to ORCHIDEE‐SOM for representing the vertical profile of soil δ^13^C, and evaluated the resultant module against δ^13^C observations from four temperate sites: a deciduous forest, a grassland and two experimental sites with C3/C4 crop transitions. The new soil module has shown itself capable of accounting for the Suess effect and CO_2_ fertilization, root versus shoot ^13^C enrichment, and ^13^C discrimination associated with heterotrophic respiration. δ^13^C model‐data comparison indicate that the model performed better when simulating the shift in the isotopic signal due to short‐term vegetation cover changes (C3 to C4) as compared with simulating the observed stock of ^13^C (arising from a combination of long‐term processes). We tested the processes individually to assess their relative importance; however, the model underestimates the vertical variation in of δ^13^C across the soil column. In addition, since the different carbon pools in the model (active, slow passive) have different δ^13^C signatures, successful representation of their relative proportions over the entire profile is key to future model evaluation. Finally, our results seem to provide strong indications that point towards the importance of long‐term land cover history in generating real and simulated vertical profiles of δ^13^C, particularly in deep soil (>1 m). This hampers the accurate simulation of observed stocks of ^13^C by a global model. This new ^13^C soil module is a first and necessary step towards the integration of stable carbon isotopes as a tool for diagnosing and improving SOC models at the global scale. We should, however, also consider the vegetative ^13^C cycle in models to better account for the Suess effect and the δ^13^C response to climate change in different plant tissues. Further work is also needed to propose a more mechanistic description of microbial ^13^C discrimination.

## Supporting information

Supporting Information S1

## References

[jame21017-bib-0001] Abramoff, R. , Xu, X. , Hartman, M. , O'Brien, S. , Feng, W. , Davidson, E. , Finzi, A. , Moorhead, D. , Schimel, J. , Torn, M. , & Mayes, M. A. (2017). The Millennial model: In search of measurable pools and transformations for modeling soil carbon in the new century. Biogeochemistry, 137(1‐2), 51–71. 10.1007/s10533-017-0409-7

[jame21017-bib-0002] Acton, P. , Fox, J. , Campbell, E. , Rowe, H. , & Wilkinson, M. (2013). Carbon isotopes for estimating soil decomposition and physical mixing in well‐drained forest soils. Journal of Geophysical Research: Biogeosciences, 118, 1532–1545. 10.1002/2013JG002400

[jame21017-bib-0003] Ågren, G. I. , Bosatta, E. , & Balesdent, J. (1996). Isotope discrimination during decomposition of organic matter: A theoretical analysis. Soil Science Society of America Journal, 60(4), 1121–1126.

[jame21017-bib-0004] Baisden, W. T. , Amundson, R. , Brenner, D. L. , Cook, A. C. , Kendall, C. , & Harden, J. W. (2002). A multiisotope C and N modeling analysis of soil organic matter turnover and transport as a function of soil depth in a California annual grassland soil chronosequence. Global Biogeochemical Cycles, 16(4), 1135 10.1029/2001GB001823

[jame21017-bib-0005] Balesdent, J. , Basile‐Doelsch, I. , Chadoeuf, J. , Cornu, S. , Derrien, D. , Fekiacova, Z. , & Hatté, C. (2018a). Atmosphere–soil carbon transfer as a function of soil depth. Nature, 559(7715), 599–602. 10.1038/s41586-018-0328-3 29995858

[jame21017-bib-0006] Balesdent, J. , Basile‐Doelsch, I. , Chadoeuf, J. , Cornu, S. , Derrien, D. , Fekiacova, Z. , & Hatté, C. (2018b). Depth distribution of soil carbon age inferred from natural 13C labelling meta‐analysis, doi:10.15454/KMNR6R. 29995858

[jame21017-bib-0007] Balesdent, J. , Girardin, C. , & Mariotti, A. (1993). Site‐related δ13C of tree leaves and soil organic matter in a temperate forest. Ecology, 74(6), 1713–1721. 10.2307/1939930

[jame21017-bib-0008] Balesdent, J. , & Mariotti, A. (1987). Natural 13C abundance as a tracer for studies of soil organic matter dynamics. Soil Biology and Biochemistry, 19(1), 25–30. 10.1016/0038-0717(87)90120-9

[jame21017-bib-0009] Balesdent, J. , Mariotti, A. , & Boisgontier, D. (1990). Effect of tillage on soil organic carbon mineralization estimated from 13C abundance in maize fields. Journal of Soil Science, 41(4), 587–596. 10.1111/j.1365-2389.1990.tb00228.x

[jame21017-bib-0010] Barré, P. , Eglin, T. , Christensen, B. T. , Ciais, P. , Houot, S. , Kätterer, T. , van Oort, F. , Peylin, P. , Poulton, P. R. , Romanenkov, V. , & Chenu, C. (2010). Quantifying and isolating stable soil organic carbon using long‐term bare fallow experiments. Biogeosciences, 7(11), 3839–3850. 10.5194/bg-7-3839-2010

[jame21017-bib-0011] Bird, M. , Kracht, O. , Derrien, D. , & Zhou, Y. (2003). The effect of soil texture and roots on the stable carbon isotope composition of soil organic carbon. Soil Research, 41(1), 77–94. 10.1071/SR02044

[jame21017-bib-0012] Bowling, D. R. , Pataki, D. E. , & Randerson, J. T. (2008). Carbon isotopes in terrestrial ecosystem pools and CO2 fluxes. The New Phytologist, 178(1), 24–40. 10.1111/j.1469-8137.2007.02342.x 18179603

[jame21017-bib-0013] Braakhekke, M. , Beer, C. , Schrumpf, M. , Ekici, A. , Ahrens, B. , Hoosbeek, M. R. , Kruijt, B. , Kabat, P. , & Reichstein, M. (2014). The use of radiocarbon to constrain current and future soil organic matter turnover and transport in a temperate forest. Journal of Geophysical Research: Biogeosciences, 119, 372–391. 10.1002/2013JG002420

[jame21017-bib-0014] Brüggemann, N. , Gessler, A. , Kayler, Z. , Keel, S. G. , Badeck, F. , Barthel, M. , Boeckx, P. , Buchmann, N. , Brugnoli, E. , Esperschütz, J. , Gavrichkova, O. , Ghashghaie, J. , Gomez‐Casanovas, N. , Keitel, C. , Knohl, A. , Kuptz, D. , Palacio, S. , Salmon, Y. , Uchida, Y. , & Bahn, M. (2011). Carbon allocation and carbon isotope fluxes in the plant‐soil‐atmosphere continuum: A review. Biogeosciences, 8(11), 3457–3489. 10.5194/bg-8-3457-2011

[jame21017-bib-0015] Brugnoli, E. , & Farquhar, G. D. (2000). Photosynthetic fractionation of carbon isotopes In Leegood R. C., Sharkey T. D., & von Caemmerer S. (Eds.), Photosynthesis: Physiology and Metabolism (pp. 399–434). Dordrecht: Springer Netherlands.

[jame21017-bib-0016] Brugnoli, E. , Hubick, K. T. , von Caemmerer, S. , Wong, S. C. , & Farquhar, G. D. (1988). Correlation between the carbon isotope discrimination in leaf starch and sugars of C(3) plants and the ratio of intercellular and atmospheric partial pressures of carbon dioxide. Plant Physiology, 88(4), 1418–1424. 10.1104/pp.88.4.1418 16666476 PMC1055774

[jame21017-bib-0017] Camino‐Serrano, M. , Guenet, B. , Luyssaert, S. , Ciais, P. , Bastrikov, V. , de Vos, B. , Gielen, B. , Gleixner, G. , Jornet‐Puig, A. , Kaiser, K. , Kothawala, D. , Lauerwald, R. , Peñuelas, J. , Schrumpf, M. , Vicca, S. , Vuichard, N. , Walmsley, D. , & Janssens, I. A. (2018). ORCHIDEE‐SOM: Modeling soil organic carbon (SOC) and dissolved organic carbon (DOC) dynamics along vertical soil profiles in Europe. Geoscientific Model Development, 11(3), 937–957. 10.5194/gmd-11-937-2018

[jame21017-bib-0018] Campoy, A. , Ducharne, A. , Cheruy, F. , Hourdin, F. , Polcher, J. , & Dupont, J. C. (2013). Response of land surface fluxes and precipitation to different soil bottom hydrological conditions in a general circulation model. Journal of Geophysical Research: Atmospheres, 118, 10725–10739. 10.1002/Jgrd.50627

[jame21017-bib-0019] Cerling, T. E. , Quade, J. , Wang, Y. , & Bowman, J. R. (1989). Carbon isotopes in soils and palaeosols as ecology and palaeoecology indicators. Nature, 341(6238), 138–139. 10.1038/341138a0

[jame21017-bib-0020] Chen, J. , Zhu, Q. , Riley, W. J. , He, Y. , Randerson, J. T. , & Trumbore, S. (2019). Comparison with global soil radiocarbon observations indicates needed carbon cycle improvements in the E3SM land model. Journal of Geophysical Research: Biogeosciences, 124, 1098–1114. 10.1029/2018JG004795

[jame21017-bib-0021] Coleman, K. , Jenkinson, D. S. , Crocker, G. J. , Grace, P. R. , Klír, J. , Körschens, M. , Poulton, P. R. , & Richter, D. D. (1997). Simulating trends in soil organic carbon in long‐term experiments using RothC‐26.3. Geoderma, 81(1–2), 29–44. 10.1016/S0016-7061(97)00079-7

[jame21017-bib-0022] Desjardins, T. , Folgarait, P. J. , Pando‐Bahuon, A. , Girardin, C. , & Lavelle, P. (2006). Soil organic matter dynamics along a rice chronosequence in north‐eastern Argentina: Evidence from natural13C abundance and particle size fractionation. Soil Biology and Biochemistry, 38(9), 2753–2761. 10.1016/j.soilbio.2006.04.029

[jame21017-bib-0023] Diefendorf, A. F. , Mueller, K. E. , Wing, S. L. , Koch, P. L. , & Freeman, K. H. (2010). Global patterns in leaf 13C discrimination and implications for studies of past and future climate. Proceedings of the National Academy of Sciences, 107(13), 5738–5743. 10.1073/pnas.0910513107 PMC2851872 20231481

[jame21017-bib-0024] Diels, J. , Vanlauwe, B. , Van Der Meersch, M. K. , Sanginga, N. , & Merckx, R. (2004). Long‐term soil organic carbon dynamics in a subhumid tropical climate: 13 C data in mixed C 3/C 4 cropping and modeling with ROTHC. Soil Biology and Biochemistry, 36, 1739–1750.

[jame21017-bib-0025] Dignac, M.‐F. F. , Bahri, H. , Rumpel, C. , Rasse, D. P. P. , Bardoux, G. , Balesdent, J. , Girardin, C. , Chenu, C. , & Mariotti, A. (2005). Carbon‐13 natural abundance as a tool to study the dynamics of lignin monomers in soil: An appraisal at the Closeaux experimental field (France). Geoderma, 128(1–2), 3–17. 10.1016/j.geoderma.2004.12.022

[jame21017-bib-0026] Duarte, H. F. , Raczka, B. M. , Ricciuto, D. M. , Lin, J. C. , Koven, C. D. , Thornton, P. E. , Bowling, D. R. , Lai, C.‐T. , Bible, K. J. , & Ehleringer, J. R. (2017). Evaluating the community land model (CLM4.5) at a coniferous forest site in northwestern United States using flux and carbon‐isotope measurements. Biogeosciences, 14(18), 4315–4340. 10.5194/bg-14-4315-2017

[jame21017-bib-0027] Ehleringer, J. R. , Buchmann, N. , & Flanagan, L. B. (2000). Carbon isotope ratios in belowground carbon cycle processes. Ecological Applications, 10(2), 412–422.

[jame21017-bib-0028] Elliott, E. T. , Paustian, K. , & Frey, S. D. (1996). Modeling the measurable or measuring the modelable: A hierarchical approach to isolating meaningful soil organic matter fractionations In Evaluation of Soil Organic Matter Models: Using Existing Long‐Term Datasets (Vol. 1994, pp. 161–179). Berlin, Heidelberg: Springer

[jame21017-bib-0029] Evrard, O. , Nord, G. , Cerdan, O. , Souchère, V. , Le Bissonnais, Y. , & Bontè, P. (2010). Modelling the impact of land use change and rainfall seasonality on sediment export from an agricultural catchment of the northwestern European loess belt. Agriculture, Ecosystems and Environment, 138(1–2), 83–94. 10.1016/j.agee.2010.04.003

[jame21017-bib-0031] Feng, X. , & Epstein, S. (1995). Carbon isotopes of trees from arid environments and implications for reconstructing atmospheric CO2 concentration. Geochimica et Cosmochimica Acta, 59(12), 2599–2608. 10.1016/0016-7037(95)00152-2

[jame21017-bib-0032] Fontaine, S. , Barot, S. , Barré, P. , Bdioui, N. , Mary, B. , & Rumpel, C. (2007). Stability of organic carbon in deep soil layers controlled by fresh carbon supply. Nature, 450(7167), 277–280. 10.1038/nature06275 17994095

[jame21017-bib-0033] Futter, M. N. , Butterfield, D. , Cosby, B. J. , Dillon, P. J. , Wade, A. J. , & Whitehead, P. G. (2007). Modeling the mechanisms that control in‐stream dissolved organic carbon dynamics in upland and forested catchments. Water Resources Research, 43, W02424 10.1029/2006WR004960

[jame21017-bib-0034] Garten, C. T. , Cooper, L. W. , Post, W. M. , & Hanson, P. J. (2000). Climate controls on forest soil C istotope ratios in the southern Appalachian mountains. Ecology, 81(4), 1108–1119. 10.1890/0012-9658(2000)081(1108:CCOFSC)2.0.CO;2

[jame21017-bib-0035] Garten, C. T. , Hanson, P. J. , Todd, D. E. , Lu, B. B. , & Brice, D. J. (2008). Natural15N‐ and13C‐abundance as indicators of forest nitrogen status and soil carbon dynamics In Stable Isotopes in Ecology and Environmental Science (pp. 61–82). Oxford, UK: Blackwell Publishing Ltd.

[jame21017-bib-0036] Gleixner, G. , Danier, H. J. , Werner, R. A. , & Schmidt, H. L. (1993). Correlations between the 13C content of primary and secondary plant products in different cell compartments and that in decomposing basidiomycetes. Plant Physiology, 102, 1287–1290. 10.1104/pp.102.4.1287 12231905 PMC158917

[jame21017-bib-0037] Gleixner, G. , Ehleringer, J. R. , & Pataki, D. E. (2005). 3 ‐ Stable isotope composition of soil organic matter A2 In Flanagan L. B. (Ed.), Stable Isotopes and Biosphere Atmosphere Interactions (pp. 29–46). San Diego, CA: Academic Press.

[jame21017-bib-0088] Graven, H. , Allison, C. , Etheridge, D. , Hammer, S. , Keeling, R. , Levin, I. , Meijer, H. A. J. , Rubino, M. , Tans, P. , Trudinger, R. C. , Vaughn, B. , & White, J. (2017). Compiled records of carbon isotopes in atmospheric CO_2_ for historical simulations in CMIP6. Geoscientific Model Development, 10(12), 4405–4417. 10.5194/gmd-10-4405-2017

[jame21017-bib-0038] Guenet, B. , Eglin, T. , Vasilyeva, N. , Peylin, P. , Ciais, P. , & Chenu, C. (2013). The relative importance of decomposition and transport mechanisms in accounting for soil organic carbon profiles. Biogeosciences, 10(4), 2379–2392. 10.5194/bg-10-2379-2013

[jame21017-bib-0039] Guenet, B. , Juarez, S. , Bardoux, G. , Luc, A. , & Claire, C. (2012). Evidence that stable C is as vulnerable to priming effect as is more labile C in soil. Soil Biology and Biochemistry, 52, 43–48. 10.1016/j.soilbio.2012.04.001

[jame21017-bib-0040] Hashimoto, S. , Carvalhais, N. , Ito, A. , Migliavacca, M. , Nishina, K. , & Reichstein, M. (2015). Global spatiotemporal distribution of soil respiration modeled using a global database. Biogeosciences, 12(13), 4121–4132. 10.5194/bg-12-4121-2015

[jame21017-bib-0041] He, Y. , Trumbore, S. E. , Torn, M. S. , Harden, J. W. , Vaughn, L. J. S. , Allison, S. D. , & Randerson, J. T. (2016). Radiocarbon constraints imply reduced carbon uptake by soils during the 21st century. Science, 353(6306), 1419–1424. 27708036 10.1126/science.aad4273

[jame21017-bib-0042] Hobbie, E. A. , & Werner, R. A. (2004). Intramolecular, compound‐specific, and bulk carbon isotope patterns in C 3 and C 4 plants: A review and synthesis. The New Phytologist, 161(2), 371–385. 10.1111/j.1469-8137.2004.00970.x 33873514

[jame21017-bib-0043] Högberg, P. , Ekblad, A. , Nordgren, A. , Plamboeck, A. H. , Ohlsson, A. , Bhupinderpal, S. , HÃgberg, M. N. , Ehleringer, J. R. , & Pataki, D. E. (2005). 4 ‐ Factors determining the 13C abundance of soil‐respired CO_2_ in Boreal Forests A2 In Flanagan L. B. (Ed.), Stable Isotopes and Biosphere Atmosphere Interactions (pp. 47–68). San Diego, CA: Academic Press.

[jame21017-bib-0044] Huang, Y. , Guenet, B. , Ciais, P. , Janssens, I. A. , Soong, J. L. , Wang, Y. , Goll, D. , Blagodatskaya, E. , & Huang, Y. (2018). ORCHIMIC (v1. 0), A microbe‐driven model for soil organic matter decomposition designed for large‐scale applications. Geoscientific Model Development, 11, 2111–2138. 10.5194/gmd-11-2111-2018

[jame21017-bib-0045] Hugh, G. , Gauch, J. Jr. , Hwang, G. , & Fick, G. W. (2003). Model evaluation by comparison of model‐based predictions and measured values. Agronomy Journal, 95(6), 1442–1446. 10.2134/agronj2003.1442

[jame21017-bib-0046] Huntzinger, D. N. , Michalak, A. M. , Schwalm, C. , Ciais, P. , King, A. W. , Fang, Y. , Schaefer, K. , Wei, Y. , Cook, R. B. , Fisher, J. B. , Hayes, D. , Huang, M. , Ito, A. , Jain, A. K. , Lei, H. , Lu, C. , Maignan, F. , Mao, J. , Parazoo, N. , Peng, S. , Poulter, B. , Ricciuto, D. , Shi, X. , Tian, H. , Wang, W. , Zeng, N. , & Zhao, F. (2017). Uncertainty in the response of terrestrial carbon sink to environmental drivers undermines carbon‐climate feedback predictions. Scientific Reports, 7(1), 4765 10.1038/s41598-017-03818-2 28684755 PMC5500546

[jame21017-bib-0047] Jagercikova, M. , Cornu, S. , Bourlès, D. , Evrard, O. , Hatté, C. , & Balesdent, J. (2017). Quantification of vertical solid matter transfers in soils during pedogenesis by a multi‐tracer approach. Journal of Soils and Sediments, 17(2), 408–422. 10.1007/s11368-016-1560-9

[jame21017-bib-0048] Jagercikova, M. , Evrard, O. , Balesdent, J. , Lefèvre, I. , & Cornu, S. (2014). Modeling the migration of fallout radionuclides to quantify the contemporary transfer of fine particles in Luvisol profiles under different land uses and farming practices. Soil and Tillage Research, 140, 82–97. 10.1016/j.still.2014.02.013

[jame21017-bib-0049] Kalbitz, K. , Schwesig, D. , Rethemeyer, J. , & Matzner, E. (2005). Stabilization of dissolved organic matter by sorption to the mineral soil. Soil Biology and Biochemistry, 37(7), 1319–1331. 10.1016/j.soilbio.2004.11.028

[jame21017-bib-0050] Kalnay, E. , Kanamitsu, M. , Kistler, R. , Collins, W. , Deaven, D. , Gandin, L. , Iredell, M. , Saha, S. , White, G. , Woollen, J. , Zhu, Y. , Leetmaa, A. , Reynolds, R. , Chelliah, M. , Ebisuzaki, W. , Higgins, W. , Janowiak, J. , Mo, K. C. , Ropelewski, C. , Wang, J. , Jenne, R. , & Joseph, D. (1996). The NCEP/NCAR 40‐year reanalysis project. Bulletin of the American Meteorological Society, 77(3), 437–471. 10.1175/1520-0477(1996)077<0437:TNYRP>2.0.CO;2

[jame21017-bib-0051] Keeling, C. D. , & Whorf, T. P. (2006). Atmospheric CO2 records from sites in the SIO air sampling network. Oak Ridge, Tenn: Oak Ridge Natl. Lab. U.S. Dept. or Energy.

[jame21017-bib-0052] Keeling, R. F. , Graven, H. D. , Welp, L. R. , Resplandy, L. , Bi, J. , Piper, S. C. , Sun, Y. , Bollenbacher, A. , & Meijer, H. A. J. (2017). Atmospheric evidence for a global secular increase in carbon isotopic discrimination of land photosynthesis. Proceedings of the National Academy of Sciences, 201619240 10.1073/pnas.1619240114 PMC5625891 28893986

[jame21017-bib-0053] Keyvanshokouhi, S. , Cornu, S. , Samouëlian, A. , & Finke, P. (2016). Evaluating SoilGen2 as a tool for projecting soil evolution induced by global change. Science of the Total Environment, 571, 110–123. 10.1016/j.scitotenv.2016.07.119 27470670

[jame21017-bib-0054] Klumpp, K. , Schäufele, R. , Lötscher, M. , Lattanzi, F. A. , Feneis, W. , & Schnyder, H. (2005). C‐isotope composition of CO2 respired by shoots and roots: Fractionation during dark respiration? Plant, Cell and Environment, 28(2), 241–250. 10.1111/j.1365-3040.2004.01268.x

[jame21017-bib-0055] Kothawala, D. N. , Moore, T. R. , & Hendershot, W. H. (2008). Adsorption of dissolved organic carbon to mineral soils: A comparison of four isotherm approaches. Geoderma, 148(1), 43–50. 10.1016/j.geoderma.2008.09.004

[jame21017-bib-0056] Koven, C. D. , Riley, W. J. , Subin, Z. M. , Tang, J. Y. , Torn, M. S. , Collins, W. D. , Bonan, G. B. , Lawrence, D. M. , & Swenson, S. C. (2013). The effect of vertically resolved soil biogeochemistry and alternate soil C and N models on C dynamics of CLM4. Biogeosciences, 10(11), 7109–7131. 10.5194/bg-10-7109-2013

[jame21017-bib-0057] Lawrence, C. R. , Beem‐Miller, J. , Hoyt, A. M. , Monroe, G. , Sierra, C. A. , Stoner, S. , Heckman, K. , Blankinship, J. C. , Crow, S. E. , McNicol, G. , Trumbore, S. , Levine, P. A. , Vindušková, O. , Todd‐Brown, K. , Rasmussen, C. , Hicks Pries, C. E. , Schädel, C. , McFarlane, K. , Doetterl, S. , Hatté, C. , He, Y. , Treat, C. , Harden, J. W. , Torn, M. S. , Estop‐Aragonés, C. , Asefaw Berhe, A. , Keiluweit, M. , Marin‐Spiotta, E. , Plante, A. F. , Thomson, A. , Schimel, J. P. , Vaughn, L. J. S. , & Wagai, R. (2019). An open source database for the synthesis of soil radiocarbon data: ISRaD Version 1.0. Earth System Science Data Discussions, 1–37. 10.5194/essd-2019-55

[jame21017-bib-0058] Lichtfouse, É. , Chenu, C. , Baudin, F. , Leblond, C. , Da Silva, M. , Behar, F. , Derenne, S. , Largeau, C. , Wehrung, P. , & Albrecht, P. (1998). A novel pathway of soil organic matter formation by selective preservation of resistant straight‐chain biopolymers: Chemical and isotope evidence. Organic Geochemistry, 28(6), 411–415. 10.1016/S0146-6380(98)00005-9

[jame21017-bib-0059] Lichtfouse, É. , Dou, S. , Girardin, C. , Grably, M. , Balesdent, J. , Behar, F. , & Vandenbroucke, M. (1995). Unexpected13C‐enrichment of organic components from wheat crop soils: Evidence for the in situ origin of soil organic matter. Organic Geochemistry, 23(9), 865–868. 10.1016/0146-6380(95)80009-G

[jame21017-bib-0060] Luo, Y. , Ahlström, A. , Allison, S. D. , Batjes, N. H. , Brovkin, V. , Carvalhais, N. , Chappell, A. , Ciais, P. , Davidson, E. A. , Finzi, A. , Georgiou, K. , Guenet, B. , Hararuk, O. , Harden, J. W. , He, Y. , Hopkins, F. , Jiang, L. , Koven, C. , Jackson, R. B. , Jones, C. D. , Lara, M. J. , Liang, J. , McGuire, A. D. , Parton, W. , Peng, C. , Randerson, J. T. , Salazar, A. , Sierra, C. A. , Smith, M. J. , Tian, H. , Todd‐Brown, K. E. O. , Torn, M. , van Groenigen, K. J. , Wang, Y. P. , West, T. O. , Wei, Y. , Wieder, W. R. , Xia, J. , Xu, X. , Xu, X. , & Zhou, T. (2016). Toward more realistic projections of soil carbon dynamics by Earth system models. Global Biogeochemical Cycles, 30, 40–56. 10.1002/2015GB005239

[jame21017-bib-0061] MacBean, N. , Peylin, P. , Chevallier, F. , Scholze, M. , & Schürmann, G. (2016). Consistent assimilation of multiple data streams in a carbon cycle data assimilation system. Geoscientific Model Development, 9, 3569–3588. 10.5194/gmd-9-3569-2016

[jame21017-bib-0062] Menichetti, L. , Houot, S. , van Oort, F. , Kätterer, T. , Christensen, B. T. , Chenu, C. , Barré, P. , Vasilyeva, N. A. , & Ekblad, A. (2015). Increase in soil stable carbon isotope ratio relates to loss of organic carbon: Results from five long‐term bare fallow experiments. Oecologia, 177(3), 811–821. 10.1007/s00442-014-3114-4 25344418

[jame21017-bib-0063] Menichetti, L. , Katterer, T. , & Leifeld, J. (2016). Parametrization consequences of constraining soil organic matter models by total carbon and radiocarbon using long‐term field data. Biogeosciences, 13(10), 3003–3019. 10.5194/bg-13-3003-2016

[jame21017-bib-0064] Mitchell, T. D. , Carter, T. R. , Jones, P. D. , Hulme, M. , & New, M. (2004). A comprehensive set of high‐resolution grids of monthly climate for Europe and the globe: The observed record (1901–2000) and 16 scenarios (2001–2100), *… Cent. Clim. …* , (July), 1–30.

[jame21017-bib-0065] Molina, J. A. E. , Clapp, C. E. , Linden, D. R. , Allmaras, R. R. , Layese, M. F. , Dowdy, R. H. , & Cheng, H. H. (2001). Modeling the incorporation of corn (Zea mays L.) carbon from roots and rhizodeposition into soil organic matter. Soil Biology and Biochemistry, 33(1), 83–92. 10.1016/S0038-0717(00)00117-6

[jame21017-bib-0066] Nachtergaele, F. , van Velthuizen, H. , van Engelen, V. , Fischer, G. , Jones, A. , Montanarella, L. , Petri, M. , Prieler, S. , Teixeira, E. , & Shi, X. (2012). Harmonized world soil database (version 1.2), *FAO, Rome, Italy IIASA, Laxenburg, Austria*, 1–50.

[jame21017-bib-0067] Navarrete, D. , Sitch, S. , Aragão, L. E. O. C. , & Pedroni, L. (2016). Conversion from forests to pastures in the Colombian Amazon leads to contrasting soil carbon dynamics depending on land management practices. Global Change Biology, 22(10), 3503–3517. 10.1111/gcb.13266 26929394

[jame21017-bib-0068] Nodvin, S. C. , Driscoll, C. T. , & Likens, G. E. (1986). Simple partitioning of anions and dissolved organic‐carbon in a forest soil. Soil Science, 142(1), 27–35. 10.1097/00010694-198607000-00005

[jame21017-bib-0069] O'Leary, M. H. (1981). Carbon isotope fractionation in plants. Phytochemistry, 20(4), 553–567.

[jame21017-bib-0070] Parton, W. J. , Schimel, D. S. , Cole, C. V. , & Ojima, D. S. (1987). Analysis of factors controlling soil organic‐matter levels in great‐plains grasslands. Soil Science Society of America Journal, 51(5), 1173–1179.

[jame21017-bib-0071] Poage, M. A. , & Feng, X. (2004). A theoretical analysis of steady state δ ^13^ C profiles of soil organic matter. Global Biogeochemical Cycles, 18, GB2016 10.1029/2003GB002195

[jame21017-bib-0072] Qiu, C. , Zhu, D. , Ciais, P. , Guenet, B. , Krinner, G. , Peng, S. , Aurela, M. , Bernhofer, C. , Brümmer, C. , Bret‐Harte, S. , Chu, H. , Chen, J. , Desai, A. R. , Dušek, J. , Euskirchen, E. S. , Fortuniak, K. , Flanagan, L. B. , Friborg, T. , Grygoruk, M. , Gogo, S. , Grünwald, T. , Hansen, B. U. , Holl, D. , Humphreys, E. , Hurkuck, M. , Kiely, G. , Klatt, J. , Kutzbach, L. , Largeron, C. , Laggoun‐Défarge, F. , Lund, M. , Lafleur, P. M. , Li, X. , Mammarella, I. , Merbold, L. , Nilsson, M. B. , Olejnik, J. , Ottosson‐Löfvenius, M. , Oechel, W. , Parmentier, F. J. W. , Peichl, M. , Pirk, N. , Peltola, O. , Pawlak, W. , Rasse, D. , Rinne, J. , Shaver, G. , Schmid, H. P. , Sottocornola, M. , Steinbrecher, R. , Sachs, T. , Urbaniak, M. , Zona, D. , & Ziemblinska, K. (2018). ORCHIDEE‐PEAT (revision 4596), A model for northern peatland CO2 water and energy fluxes on daily to annual scales. Geoscientific Model Development, 11(2), 497–519. 10.5194/gmd-2017-155

[jame21017-bib-0073] Raczka, B. , Duarte, H. F. , Koven, C. D. , Ricciuto, D. , Thornton, P. E. , Lin, J. C. , & Bowling, D. R. (2016). An observational constraint on stomatal function in forests: evaluating coupled carbon and water vapor exchange with carbon isotopes in the Community Land Model (CLM 4.5). Biogeosciences, 13, 5183–5204. 10.5194/bg-2016-73

[jame21017-bib-0074] Rasse, D. P. , Mulder, J. , Moni, C. , & Chenu, C. (2006). Carbon turnover kinetics with depth in a French loamy soil. Soil Science Society of America Journal, 70, 2097–2105.

[jame21017-bib-0075] Schmidt, M. W. I. , Torn, M. S. , Abiven, S. , Dittmar, T. , Guggenberger, G. , Janssens, I. A. , Kleber, M. , Kögel‐Knabner, I. , Lehmann, J. , Manning, D. A. C. , Nannipieri, P. , Rasse, D. P. , Weiner, S. , & Trumbore, S. E. (2011). Persistence of soil organic matter as an ecosystem property. Nature, 478(7367), 49–56. 10.1038/nature10386 21979045

[jame21017-bib-0076] Schrumpf, M. , Kaiser, K. , Guggenberger, G. , Persson, T. , Kögel‐Knabner, I. , & Schulze, E.‐D. (2013). Storage and stability of organic carbon in soils as related to depth, occlusion within aggregates, and attachment to minerals. Biogeosciences, 10(3), 1675–1691. 10.5194/bg-10-1675-2013

[jame21017-bib-0077] Schweizer, M. , Fear, J. , & Cadisch, G. (1999). Isotopic (13C) fractionation during plant residue decomposition and its implications for soil organic matter studies. Rapid Communications in Mass Spectrometry, 13(13), 1284–1290. 10.1002/(SICI)1097-0231(19990715)13:13<1284::AID-RCM578>3.0.CO;2-0 10407311

[jame21017-bib-0078] Tans, P. P. , De Jong, A. F. M. , & Mook, W. G. (1979). Natural atmospheric 14C variation and the Suess effect. Nature, 280(5725), 826–828. 10.1038/280826a0

[jame21017-bib-0079] Tifafi, M. , Camino‐Serrano, M. , Hatté, C. , Morras, H. , Moretti, L. , Cornu, S. , & Guenet, B. (2018). The use of radiocarbon 14C to constrain soil carbon dynamics in the land surface model ORCHIDEE. Geoscientific Model Development, 11, 4711–4726. 10.5194/gmd-2018-102

[jame21017-bib-0080] Todd‐Brown, K. E. O. , Randerson, J. T. , Post, W. M. , Hoffman, F. M. , Tarnocai, C. , Schuur, E. A. G. , & Allison, S. D. (2013). Causes of variation in soil carbon simulations from CMIP5 Earth system models and comparison with observations. Biogeosciences, 10(3), 1717–1736. 10.5194/bg-10-1717-2013

[jame21017-bib-0081] Wang, C. , Houlton, B. Z. , Liu, D. , Hou, J. , Cheng, W. , & Bai, E. (2018). Stable isotopic constraints on global soil organic carbon turnover. Biogeosciences, 15(4), 987–995. 10.5194/bg-15-987-2018

[jame21017-bib-0083] Werth, M. , & Kuzyakov, Y. (2010). 13C fractionation at the root–microorganisms–soil interface: A review and outlook for partitioning studies. Soil Biology and Biochemistry, 42(9), 1372–1384. 10.1016/j.soilbio.2010.04.009

[jame21017-bib-0084] Wordell‐Dietrich, P. , Don, A. , & Helfrich, M. (2017). Controlling factors for the stability of subsoil carbon in a Dystric Cambisol. Geoderma, 304, 40–48. 10.1016/j.geoderma.2016.08.023

[jame21017-bib-0085] Wynn, J. G. , Harden, J. W. , & Fries, T. L. (2006). Stable carbon isotope depth profiles and soil organic carbon dynamics in the lower Mississippi Basin. Geoderma, 131(1â€“2), 89–109.

[jame21017-bib-0086] Yakir, D. , & Wang, X.‐F. (1996). Fluxes of CO2 and water between terrestrial vegetation and the atmosphere estimate from isotope measurementes. Nature, 380, 515–517.

