# Supplementary material for: Including Stable Carbon Isotopes to Evaluate the Dynamics of Soil Carbon in the Land‐Surface Model ORCHIDEE
Source: J Adv Model Earth Syst. 2019 Nov 17;11(11):3650–69. doi: 10.1029/2018MS001392 (PMC6988498; doi:10.1029/2018MS001392)
Supplement: Supplementary file 1 — Supporting Information S1 [file JAME-11-3650-s001.docx]

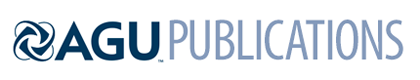


*Journal of Advances in Modeling Earth Systems*

Supporting Information for

Including stable carbon isotopes to evaluate the dynamics of soil carbon in the land-surface model ORCHIDEE

Marta Camino-Serrano^1,2^, Marwa Tifafi^3^, Jérôme Balesdent^4^, Christine Hatté^3^, Josep Peñuelas ^1,2^, Sophie Cornu^4^, Bertrand Guenet^3^

^1^CREAF, Cerdanyola del Vallès, 08193, Catalonia, Spain

^2^CSIC, Global Ecology Unit CREAF-CSIC-UAB, Bellaterra 08193, Catalonia, Spain

^3^Laboratoire des Sciences du Climat et de l’Environnement, LSCE/IPSL, CEA-CNRS-UVSQ, Université Paris-Saclay, F-91191 Gif-sur-Yvette, France.

^4^Aix Marseille Univ, CNRS, IRD, INRA, Coll France, CEREGE 13545 Aix en Provence, France

**Contents of this file**

Text S1

Figures S1 to S3

**Introduction**

In the supplementary material we present in details a description of the model and we provide three additional figures describing a sensitivity analysis to the litter δ^13^C initial value (Fig. S1), the distribution of the different pools with depth (Fig. S2) and the δ^13^C-CO_2_ values we used during our historical simulations (Fig. S3).

*Delete all unused file types below. Copy/paste for multiples of each file type as needed.*

Text S1.

**Code description**

ORCHIDEE-SOM is an extension to the soil module in ORCHIDEE, based on the ORCHIDEE version SVN r3340. In the standard version of ORCHIDEE, soil carbon is based on the CENTURY model following Parton et al., (1988) with two litter pools (metabolic and structural) and three soil organic carbon (SOC) pools (slow, active and passive), each with different decomposition rates. In addition two DOC pools were added, one considered as labile and another as recalcitrant. These two pools can be free in the soil solution or adsorbed in the mineral matrix. A fraction of the decomposed carbon is transferred from one pool to one of the free DOC and the rest is lost to the atmosphere as heterotrophic respiration. Then, the DOC can be decomposed and the C redistributed into the three SOC pools with a fraction being respired. ORCHIDEE-SOM simulate carbon dynamics in the soil column down to two meters depth, partitioned in 11 layers as described elsewhere [*Campoy et al.*, 2013; *Guimberteau et al.*, 2014]. ORCHIDEE-SOM also consider DOC concentration in each soil layer and its transport between layers. Moreover, the SOC decomposition represent priming effect with the amount of fresh organic matter as a decomposition driver [*Guenet et al.*, 2018].

## 1. Vertical discretization of the soil carbon module

ORCHIDEE-SOM represents a two-meter soil column with 11 discrete layers of geometrically increasing thicknesses with depth. For mathematical reasons, we adopted the discretization used for the soil hydrology scheme [*Campoy et al.*, 2013] to represent the C discretisation. The midpoint depths (in meters from the surface) of the layers in the discretized soil column are: 0.00098, 0.00391, 0.00978, 0.02151, 0.04497, 0.09189, 0.18573, 0.37341, 0.74878, 1.49951, respectively.

## 2. Biological and physical processes affecting SOC and DOC

### 2.1. Litter, SOC and DOC dynamics within each soil layer

In ORCHIDEE-SOM, litter is described using two pools called metabolic and structural with high and low turnover rates, respectively. Above- and belowground litter are separate pools. Belowground litter is discretized over the 11-layers scheme down to two meters, whereas aboveground litter layer is simply defined by a fixed thickness parameter. Belowground litter is distributed within the soil column following an exponential root profile with different root density profile parameter (*α*) for each PFT.

$rp=1/(1-e^{({-depth}/\alpha)})$ (1)

with *rp* being the root profile, *depth* the maximum depth of the model (fixed to two meters) and *α* a PFT parameter dependent (in meters).

Litter (*LitterC*) decomposition for each pool *i* is described by first order kinetics (Eq. 2):

$\frac{\partial{LitterC}_{i,z}}{\partial t} ={I(t)}_{i,z}-k_{LitterCi}\times{LitterC}_{i,z}(t)\times\theta(t)\times\tau(t)$ (2)

with *I* being the carbon input coming from plant death in g C m^-2^ ground days^-1^ and *k_LitterC_* the litter turnover rate constants in days^-1^, which are fixed and similar to the rates used for SOC in ORCHIDEE SVN r3340. The litter decomposition is affected by two rates modifiers, *θ* and *τ*, representing the effect of moisture and temperature, respectively:

$\theta=max(0.25,\min\left( 1,1.1\times M^{2}+2.4\times M+0.29 \right))$ (3)

$\tau=min(1,e^{0.69\times(T-303.15)/10)})$ (4)

With *M* and *T* being the soil moisture (m^3^ m^-3^) and the temperature (K) of a given layer. For the aboveground litter (dimensionless), averaged moisture and temperature over the four first layers are used to calculate the rate modifiers given by Eq. 3 and 4.

The SOC is defined by three pools (active, slow and passive) with different turnover rates. The SOC decomposition is based on *Guenet et al.*, [2013]:

$\frac{\partial{SOC}_{i,z}}{\partial t}={DOC}_{Recycled,i,j}\left( t \right)-k_{SOC,i}\times(1-e^{-c\times{LOC}_{z}(t)})\times{SOC\left( t \right)}_{i,z}\times\theta(t)\times\tau(t)$ (5)

with *DOC_recycled_* being the not-respired DOC (defined below) that is redistributed into the pool *i* considered for each soil layer *z* coming in g C m^-2^ days^-1^, *k_SOC_* a SOC turnover rate constant (days^-1^), *LOC* the stock of labile organic C defined as the sum of the C pools with a higher turnover rate than the pool considered within each soil layer *z*. We therefore considered that for the active carbon pool *LOC* is the litter and DOC, but for the slow carbon pool *LOC* is the sum of the litter, DOC and so on. Finally, *c* is a parameter controlling the impact of the *LOC* pool on the SOC mineralization rate, i.e., the priming effect [*Guenet et al.*, 2016]. Finally, the decomposition of the active SOC pool is also modulated by a clay modifier 𝛾:

$\gamma=1-0.75 x clay$ (6)

In ORCHIDEE-SOM, all the products of decomposition from litter and SOC go to free DOC, assuming that the solubilisation is a necessary for the uptake and degradation of organic matter by microorganisms [*Marschner and Kalbitz*, 2003].

In the model, DOC is represented using two pools that are defined by their turnover rates; the labile DOC pool with a high turnover rate, and the stable DOC pool with a lower turnover rate. The labile pool corresponds to the DOC coming from litter and active carbon, while the stable pool corresponds to the DOC coming from slow and passive carbon. The DOC pools in the model can be *free* in the soil solution or *adsorbed* to the soil minerals. Only the free DOC is decomposed in the model, following first order kinetics equation (Eq. 7).

$\frac{\partial{DOC}_{i,z}}{\partial t}={I_{litter}(t)}_{i,z}{{+ I}_{SOC}(t)}_{i,z}-k_{DOC,i}\times{DOC(t)}_{i,z}$ (7)

With *I_litter_* being the input coming from litter decomposition and *I_SOC_* the input coming from SOC decomposition (corresponding to the second term of equation 2 and 5, respectively) in g C m^-2^ ground days^-1^, *k_DOC_* a parameter representing the turnover rate constant of free DOC pool *i* (labile, and stable) in days^-1^. A fraction of the decomposed DOC (second term of Eq. 7) is respired whereas the other fraction is redistributed in the SOC pools following Eq. 8:

${Resp_{DOC,i,z}(t)=\left( 1-{CUE}_{DOC} \right)x k}_{DOC,i}\times{DOC(t)}_{i,z}$ (8)

The not-respired DOC (DOC_Recycled_) coming from active, slow and passive SOC pools are redistributed in the different SOC pools following the same parameters as in the CENTURY model [*Parton et al.*, 1988; *Guenet et al.*, 2016]:

${DOC}_{Recycled,i,j}(t)=fra\_carb\_ij \times{CUE}_{DOC}\times K_{DOC,i}\times{DOC(t)}_{i,z}$ (9)

With DOC_Recycled,i,j_ being the DOC flux going back from pool *i* to pool *j* and *frac_carb_ij* the prescribed fraction of carbon from pool *i* to *j*.

### 2.2. DOC sorption to soil minerals

DOC retention in mineral soils is described using the simple Initial Mass (IM) linear isotherm [*Neff and Asner*, 2001; *Wu et al.*, 2013] :

${DOC}_{RE}=m\times{DOC}_{i}-b$ (10)

With *DOC_RE_* being the amount of DOC desorbed (negative value) or adsorbed (positive value), *m* a regression coefficient similar to the partitioning coefficient, *DOC_i_* the initial concentration of free DOC in solution and *b* the intercept (the desorption parameter) in g kg^-1^ soil.

In principle, the IM and linear approaches are expressions of a simple partitioning process, where the tendency of the soil to adsorb DOC is described by an equilibrium partition coefficient (K_D_) described by Eq. (11):

$K_{D}=\frac{m}{1-m}\times\frac{(volume of solution)}{(mass of soil)}$ (11)

ORCHIDEE-SOM assumes that adsorption/desorption fluxes are controlled by the difference between the actual concentration of adsorbed DOC and the equilibrium adsorbed DOC defined by K_D._ Thus, the DOC adsorption is described as follows:

$DOC_{RE-EQ}=K_{D}\times{DOC}_{T}(t)$ (12)

$\frac{{\partial DOC}_{i}}{\partial t}={DOC}_{i}(t)-(DOC_{RE-EQ}(t)-{DOCad}_{i}(t))$ (13)

$\frac{{\partial DOCad}_{i}}{\partial t}={DOCad}_{i}(t)+(\mathrm{DOC}_{RE-EQ}(t)-{DOCad}_{i}(t))$ (14)

In Eq. 12, DOC_RE-EQ_ is the amount of adsorbed DOC in equilibrium according to the partition coefficient K_D_ (unitless). DOC_T_(t), DOC_i_(t) and DOCad_i_(t) are the total DOC (the sum of free and adsorbed DOC), the free DOC and the adsorbed DOC for each pool (labile and stable) in g C m^-2^ ground, respectively.

This approach assumes that the free DOC produced at every time step of the model (30 minutes) is immediately distributed between the adsorbed and free pools to reach equilibrium, in agreement with studies showing that sorption occurs rapidly, within seconds to minutes [*Qualls and Haines*, 1992; *Kothawala et al.*, 2008]. Finally K_D_ is controlled by soil properties following eq. 15 :

$\log K_{D}=0.001226-0.000212*pH+0.00374*Clay$ (15)

### 2.3. Vertical fluxes of SOC and DOC

ORCHIDEE-SOM assumes that SOC and DOC move along the soil profile as a result of three processes: bioturbation results in vertical fluxes of SOC, and diffusion and advection produces vertical fluxes of DOC. Bioturbation is represented using Fick’s diffusion equation [*Obrien and Stout*, 1978; *Elzein and Balesdent*, 1995; *Wynn et al.*, 2005; *Braakhekke et al.*, 2011]. The same equation is used to represent DOC diffusion but with different values for the *D* parameter.

$F_{D}= -D\times\frac{\partial^{2}C}{\partial z^{2}}$(16)

where F_D_ is the flux of C transported by diffusion in g C m^-3^ soil day^-1^, *-D* the diffusion coefficient (m^2^ day^-1^) and *C* the amount of carbon in the pool subject to transport (g C m^-3^ soil).

DOC can also be transported with the liquid phase by means of advection [*Futter et al.*, 2007; *Braakhekke et al.*, 2011]. The calculation of advection fluxes in ORCHIDEE-SOM relies on the flux of water between soil layers as calculated by the soil hydrology module and is described in eq. 17.

$F_{A}=A\times{DOC}_{i}$(17)

With F_A_ the advection flux of free DOC in g C m^-2^ 30 min^-1^, A the flux of water calculated by the hydrological module in kg m^-2^ 30 min^-1^, and DOC_i_ the concentration of DOC free in solution in pool *i* in g C m^-3^ water.

At every time step, DOC in each layer is updated with the DOC fluxes entering and leaving the soil layer. The final DOC concentration in the last and the first five layers is multiplied by drainage and runoff, respectively, to calculate the amount of DOC leaving the system (g C m^-2^ ground).

**References**

Boudreau, B. P. (1986), Mathematics of Tracer Mixing in Sediments .1. Spatially-Dependent, Diffusive Mixing, *Am. J. Sci.*, *286*(3), 161–198.

Braakhekke, M. C., C. Beer, M. R. Hoosbeek, M. Reichstein, B. Kruijt, M. Schrumpf, and P. Kabat (2011), SOMPROF: A vertically explicit soil organic matter model, *Ecol. Modell.*, *222*(10), 1712–1730, doi:DOI 10.1016/j.ecolmodel.2011.02.015.

Buckingham, E. (1907), *Studies on the Movement of Soil Moisture.*, US Government Printing Office., Washington.

Camino-Serrano, M. et al. (2014), Linking variability in soil solution dissolved organic carbon to climate, soil type, and vegetation type, *Global Biogeochem. Cycles*, 497–509, doi:10.1002/2013GB004726.Received.

Campoy, A., A. Ducharne, F. Cheruy, F. Hourdin, J. Polcher, and J. C. Dupont (2013), Response of land surface fluxes and precipitation to different soil bottom hydrological conditions in a general circulation model, *J. Geophys. Res.*, *118*(19), 10725–10739, doi:Doi 10.1002/Jgrd.50627.

d’Orgeval, T., J. Polcher, and P. de Rosnay (2008), Sensitivity of the West African hydrological cycle in ORCHIDEE to infiltration processes, *Hydrol. Earth Syst. Sci.*, *12*(6), 1387–1401.

Darcy, H. (1856), *Les fontaines de la ville de Dijon.*, Paris.

Elzein, A., and J. Balesdent (1995), Mechanistic Simulation of Vertical Distribution of Carbon Concentrations and Residence Times in Soils, *Soil Sci. Soc. Am. J.*, *59*(5), 1328–1335, doi:10.2136/sssaj1995.03615995005900050019x.

Friedli H., Liitscher H., Oeschger H., Seigenthaler U., and Stauffer B. ( 1986), Ice core record of the ^13^C/^12^C ratio of atmospheric CO, in the past two centuries. *Nature* *324*, 237-238.

Futter, M. N., D. Butterfield, B. J. Cosby, P. J. Dillon, a. J. Wade, and P. G. Whitehead (2007), Modeling the mechanisms that control in-stream dissolved organic carbon dynamics in upland and forested catchments, *Water Resour. Res.*, *43*(2), 1–16, doi:10.1029/2006WR004960.

Guenet, B., F. E. Moyano, N. Vuichard, G. J. D. Kirk, P. H. Bellamy, S. Zaehle, and P. Ciais (2013), Can we model observed soil carbon changes from a dense inventory? A case study over England and Wales using three versions of the ORCHIDEE ecosystem model (AR5, AR5-PRIM and O-CN), *Geosci. Model Dev.*, *6*(6), 2153–2163, doi:10.5194/gmd-6-2153-2013.

Guenet, B., F. E. Moyano, P. Peylin, P. Ciais, and I. A. Janssens (2016), Towards a representation of priming on soil carbon decomposition in the global land biosphere model ORCHIDEE (version 1.9.5.2), *Geosci. Model Dev.*, *9*(2), 841–855, doi:10.5194/gmd-9-841-2016.

Guenet, B., M. Camino-Serrano, P. Ciais, M. Tifafi, F. Maignan, J. L. Soong, and I. A. Janssens (2018), Impact of priming on global soil carbon stocks, *Glob. Chang. Biol.*, *24*(5), 1873–1883, doi:10.1111/gcb.14069.

Guimberteau, M., P. Ciais, A. Ducharne, J. P. Boisier, S. Peng, M. De Weirdt, and H. Verbeeck (2014), Two soil hydrology formulations of ORCHIDEE (version Trunk.rev1311) tested for the Amazon basin, *Geosci. Model Dev. Discuss.*, *7*(1), 73–129, doi:10.5194/gmdd-7-73-2014.

Jardine, P. M., N. L. Weber, and J. F. Mccarthy (1989), Mechanisms of Dissolved Organic-Carbon Adsorption on Soil, *Soil Sci. Soc. Am. J.*, *53*(5), 1378–1385.

Kaiser, K., G. Guggenberger, and W. Zech (1996), Sorption of DOM and DOM fractions to forest soils, *Geoderma*, *74*(3–4), 281–303, doi:Doi 10.1016/S0016-7061(96)00071-7.

Kothawala, D. N., T. R. Moore, and W. H. Hendershot (2008), Adsorption of dissolved organic carbon to mineral soils: A comparison of four isotherm approaches, *Geoderma*, *148*(1), 43–50, doi:10.1016/j.geoderma.2008.09.004.

Laine-Kaulio, H., H. Koivusalo, A. S. Komarov, M. Lappalainen, S. Launiainen, and A. Laurén (2014), Extending the ROMUL model to simulate the dynamics of dissolved and sorbed C and N compounds in decomposing boreal mor, *Ecol. Modell.*, *272*(0), 277–292, doi:http://dx.doi.org/10.1016/j.ecolmodel.2013.09.026.

Lauerwald, R., P. Regnier, M. Camino-Serrano, B. Guenet, M. Guimberteau, A. Ducharne, J. Polcher, and P. Ciais (2017), ORCHILEAK: A new model branch to simulate carbon transfers along the terrestrial-aquatic continuum of the Amazon basin, *Geosci. Model Dev.*, *10*, 3821–3859, doi:10.5194/gmd-2017-79.

Marschner, B., and K. Kalbitz (2003), Controls of bioavailability and biodegradability of dissolved organic matter in soils, *Geoderma*, *113*, 211–235.

McCarroll D, et al. (2009), Correction of tree ring stable carbon isotope chronologies for changes in the carbon dioxide content of the atmosphere. *Geochim Cosmochim Acta, 73*, 1539–1547

Michalzik, B., E. Tipping, J. Mulder, J. F. G. Lancho, E. Matzner, C. L. Bryant, N. Clarke, S. Lofts, and M. A. V Esteban (2003), Modelling the production and transport of dissolved organic carbon in forest soils, *Biogeochemistry*, *66*(3), 241–264.

Moore, T. R., W. Desouza, and J. F. Koprivnjak (1992), Controls on the Sorption of Dissolved Organic-Carbon by Soils, *Soil Sci.*, *154*(2), 120–129, doi:Doi 10.1097/00010694-199208000-00005.

Neff, J. C., and G. P. Asner (2001), Dissolved Organic Carbon in Terrestrial Ecosystems: Synthesis and a Model, *Ecosystems*, *4*(1), 29–48, doi:10.1007/s100210000058.

Nodvin, S. C., C. T. Driscoll, and G. E. Likens (1986), Simple Partitioning of Anions and Dissolved Organic-Carbon in a Forest Soil, *Soil Sci.*, *142*(1), 27–35, doi:Doi 10.1097/00010694-198607000-00005.

Obrien, B. J., and J. D. Stout (1978), Movement and Turnover of Soil Organic-Matter as Indicated by Carbon Isotope Measurements, *Soil Biol. Biochem.*, *10*(4), 309–317, doi:Doi 10.1016/0038-0717(78)90028-7.

Parton, W. J., J. W. B. Stewart, and C. V. Cole (1988), Dynamics of C, N, P and S in grassland soils: a model, *Biogeochemistry*, *5*(1), 109–131, doi:10.1007/BF02180320.

Qualls, R., and B. L. Haines (1992), Measuring Adsorption Isotherms Using Continuous, Unsaturated Flow through Intact Soil Cores, *Soil Sci. Soc. Am. J.*, *56*, 456–460.

de Rosnay, P., J. Polcher, M. Bruen, and K. Laval (2002), Impact of a physically based soil water flow and soil-plant interaction representation for modeling large-scale land surface processes, *J. Geophys. Res.*, *107*(D11), doi:Artn 4118Doi 10.1029/2001jd000634.

Wu, H., C. Peng, T. R. Moore, D. Hua, C. Li, Q. Zhu, M. Peichl, M. a. Arain, and Z. Guo (2013), Modeling dissolved organic carbon in temperate forest soils: TRIPLEX-DOC model development and validation, *Geosci. Model Dev. Discuss.*, *6*(2), 3473–3508, doi:10.5194/gmdd-6-3473-2013.

Wynn, J. G., M. I. Bird, and V. N. L. Wong (2005), Rayleigh distillation and the depth profile of 13C/12C ratios of soil organic carbon from soils of disparate texture in Iron Range National Park, Far North Queensland, Australia, *Geochim. Cosmochim. Acta*, *69*(8), 1961–1973.


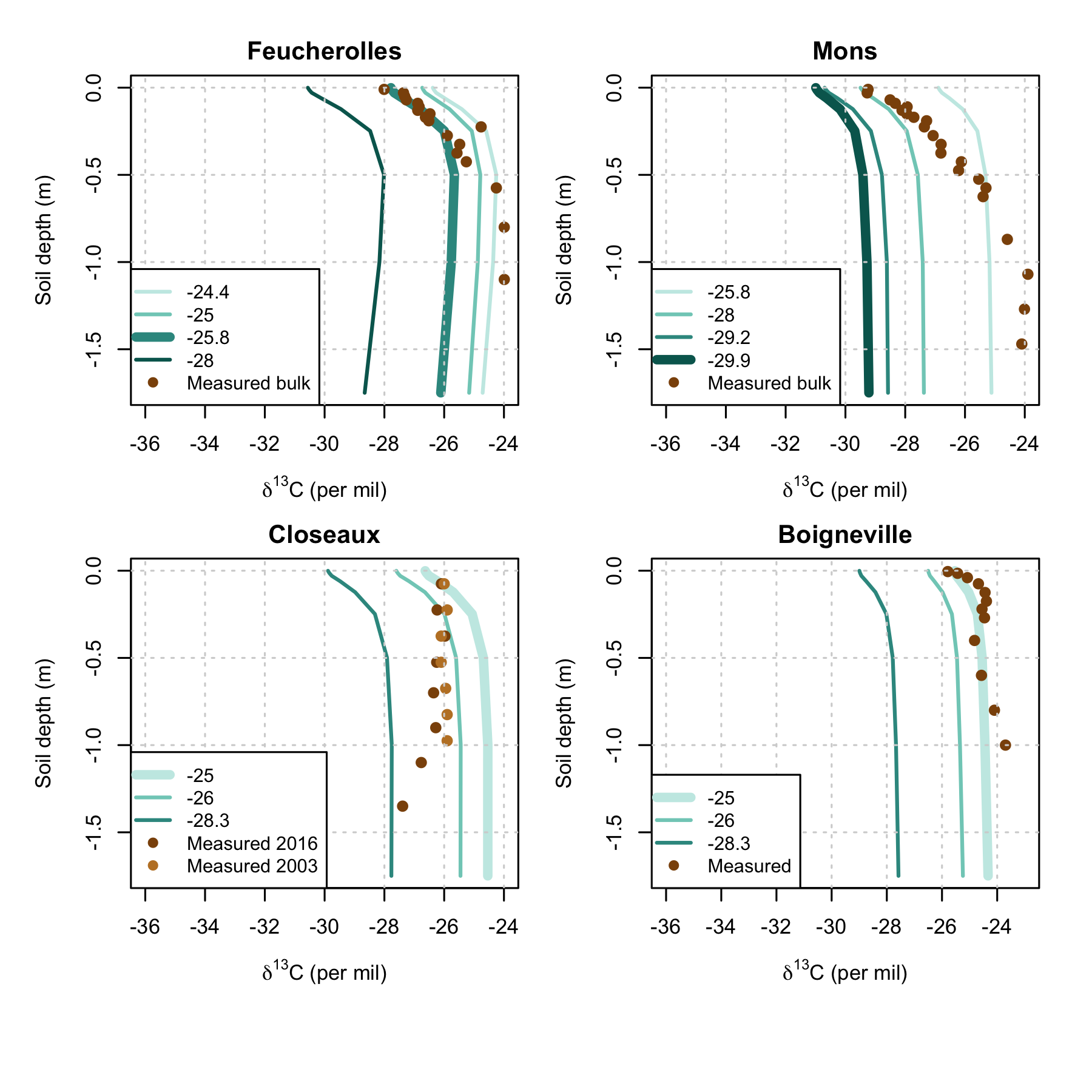


**Figure S1.** Comparison of the simulated and measured vertical soil δ^13^C profiles for the four sites using a different input litter δ^13^C for each site simulation. The data and simulations for Closeaux and Boigneville correspond to the control simulation. Note: this small sensitivity analysis used a previous version of the model without the depth-dependence of the diffusion coefficient.


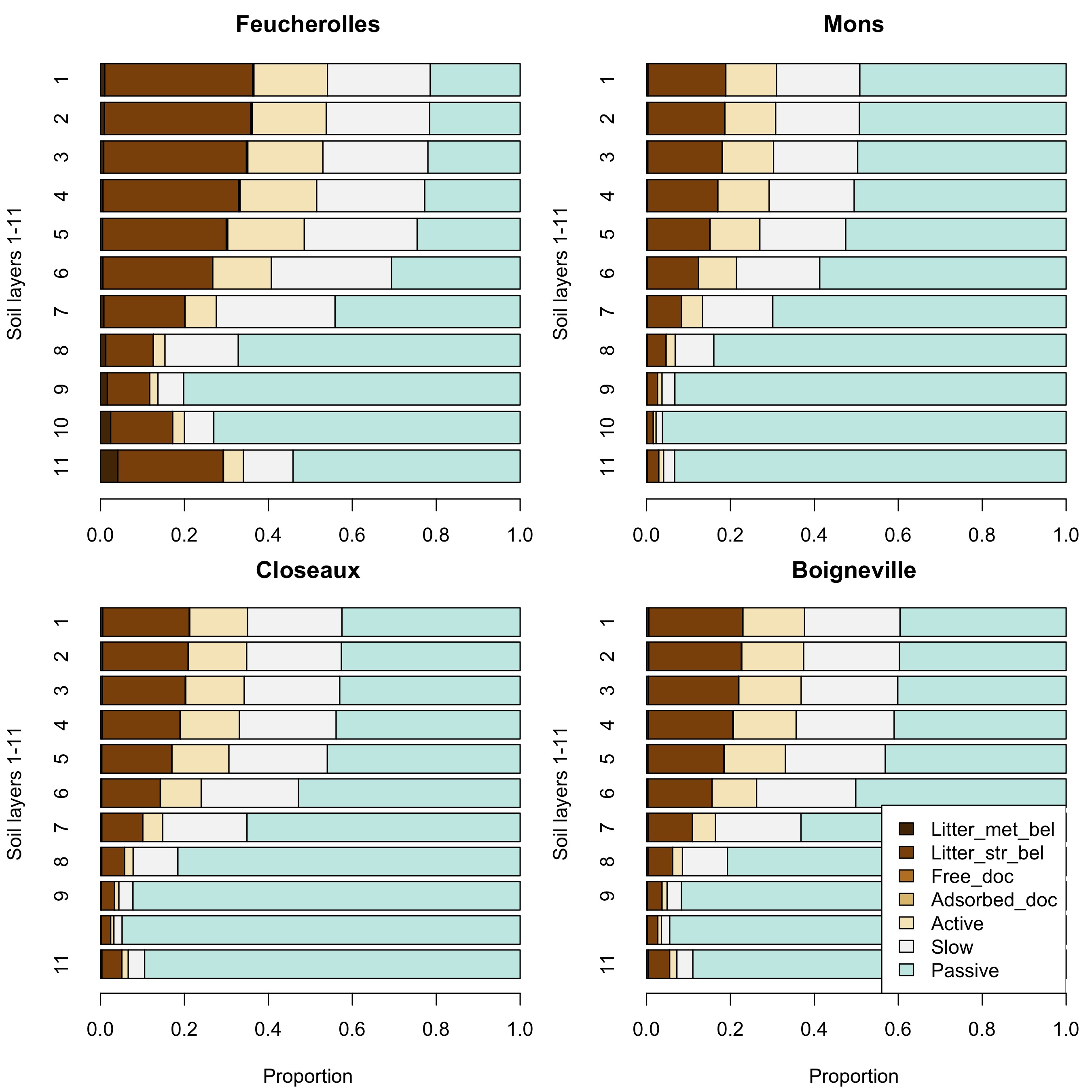


**Figure S2.** Relative proportion of each pool of soil carbon simulated by ORCHIDEE-SOM for the four sites.


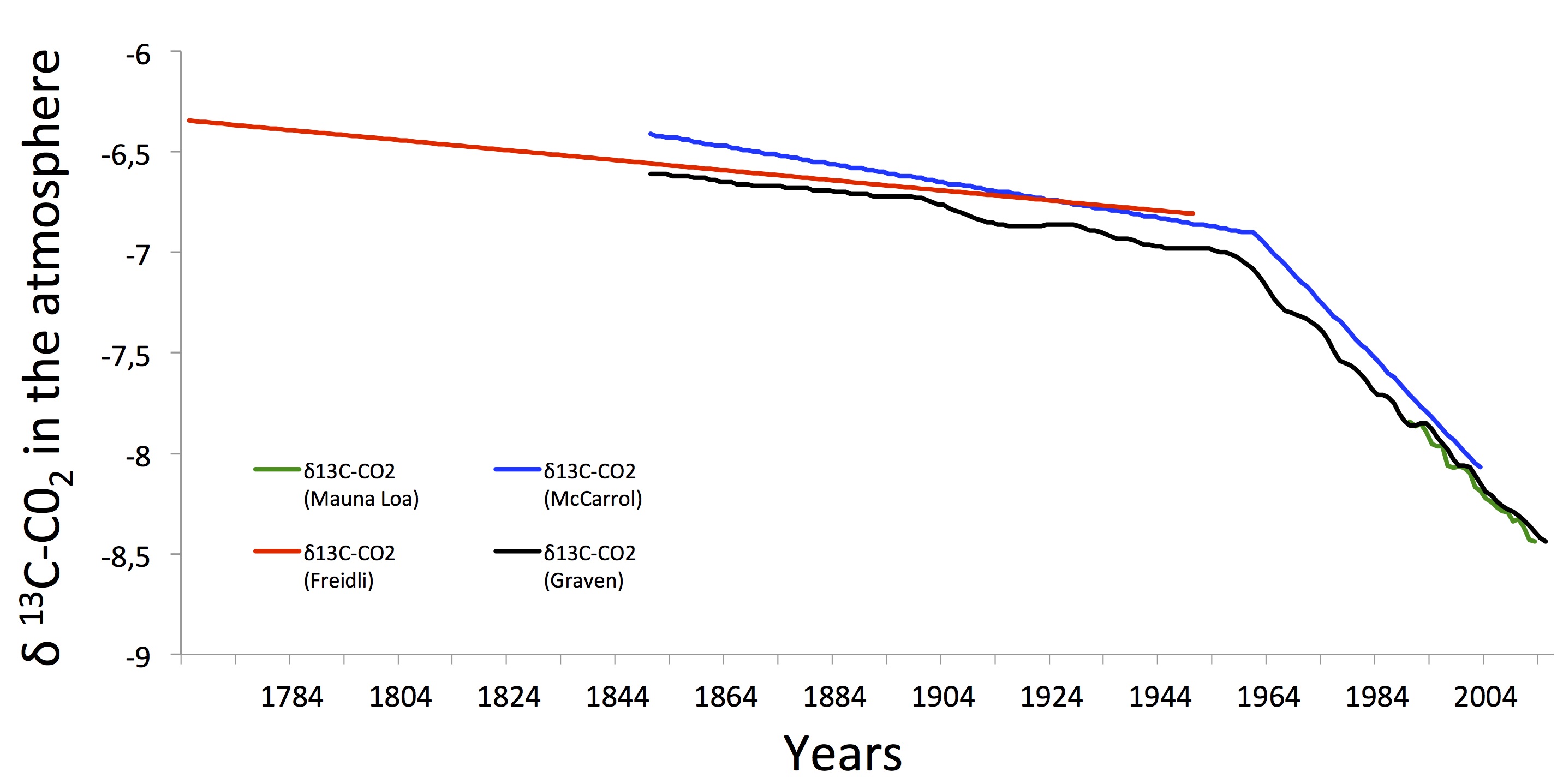


**Figure S3:** Times series of δ^13^C-CO_2_ in the atmosphere based on data records at Mauna Loa station (green line), on *McCarrol et al., [2009]*(blue line), *Freidli et al., [1986]* (red line) and *Graven et al.*, [2017] (black line)
